# Confronting an individual-based simulation model with empirical community patterns of grasslands

**DOI:** 10.1371/journal.pone.0236546

**Published:** 2020-07-28

**Authors:** Franziska Taubert, Jessica Hetzer, Julia Sabine Schmid, Andreas Huth

**Affiliations:** 1 Department of Ecological Modelling, Helmholtz Centre for Environmental Research–UFZ, Leipzig, Saxony, Germany; 2 Institute of Environmental Systems Research, University of Osnabrück, Osnabrück, Lower Saxony, Germany; 3 German Centre for Integrative Biodiversity Research (iDiv) Halle-Jena-Leipzig, Leipzig, Saxony, Germany; Irstea, FRANCE

## Abstract

Grasslands contribute to global biogeochemical cycles and can host a high number of plant species. Both–species dynamics and biogeochemical fluxes–are influenced by abiotic and biotic environmental factors, management and natural disturbances. In order to understand and project grassland dynamics under global change, vegetation models which explicitly capture all relevant processes and drivers are required. However, the parameterization of such models is often challenging. Here, we report on testing an individual- and process-based model for simulating the dynamics and structure of a grassland experiment in temperate Europe. We parameterized the model for three species and confront simulated grassland dynamics with empirical observations of their monocultures and one two-species mixture. The model reproduces general trends of vegetation patterns (vegetation cover and height, aboveground biomass and leaf area index) for the monocultures and two-species community. For example, the model simulates well an average annual grassland cover of 70% in the species mixture (observed cover of 77%), but also shows mismatches with specific observation values (e.g. for aboveground biomass). By a sensitivity analysis of the applied inverse model parameterization method, we demonstrate that multiple vegetation attributes are important for a successful parameterization while leaf area index revealed to be of highest relevance. Results of our study pinpoint to the need of improved grassland measurements (esp. of temporally higher resolution) in close combination with advanced modelling approaches.

## Introduction

Europe is covered with grasslands by nearly 21%, which is comparable with the extent of forests and crop cultivation [1]. Grasslands in the temperate zone–being an early state of succession towards forest ecosystems–are characterized predominantly by the way how they are maintained (e.g. by grazing or mowing) as well as by local environmental conditions. Different types of grasslands can emerge, ranging between extensive grasslands (e.g. for nature conservation) and intensively used meadows or pastures (e.g. for agricultural production on fertile soils) [2, 3].

In large biodiversity experiments and exploratories across various environmental gradients [4–8], a third dimension of important drivers for grassland dynamics and functioning has been analyzed–the diversity of plant species. The relations between species diversity and ecosystem functioning can have a large impact on biogeochemistry (e.g. positive effects of high richness on productivity and soil carbon storage), but also leave uncertainties for explaining variations in biodiversity effects, at the local as well as across spatial scales [5, 9, 10].

In this respect, process-based vegetation models of grasslands can complement field observations by providing additional insights into ecosystem processes and mechanisms which can be difficult to measure in the field or to infer from monitoring campaigns. For such purposes, existing grassland models differ and range from simple mathematical models (e.g. [11–13]) to more complex ones like rule-based models (e.g. [14]) or process-based individual-centred or individual-based models (IBMs, e.g. [15–21]). IBMs, in particular, explicitly represent the relevant processes for the interplay between species diversity, the environment and anthropogenic influences [22].

Here, we report on such an individual- and process-based model (GRASSMIND) which simulates the dynamics and structure of temperate grasslands including species diversity, local abiotic factors and mowing [15]. Plant growth is modeled based on the balance between carbon uptake and release, which is influenced by (a) interactions within and between species, and the plant’s response to (b) climate and soil conditions and (c) events of mowing. Notably, intra- and interspecific species interactions emerge from the interplay of species traits and individual plant size.

Model complexity often goes along with challenges. A detailed description of processes requires a larger number of parameters–from the individual plant level (e.g. species traits) to the ecosystem level (e.g. litter decomposition rates). Still, individual-based measurements of plants in species-rich grasslands are scarce which can challenge the parameterization of such a model type. Here, we show that (a) the individual-based model GRASSMIND reproduces general trends of a multiple of empirically observed patterns (time-series of aboveground biomass, leaf area index, vegetation height and cover) of three selected parameterized grassland species based on a local grassland experiment in Germany and (b) the model allows to compare simulations of monocultures and a two-species mixture with observed dynamics. We specifically ask: Can we parameterize an individual-based grassland model for different species? How much information on grasslands is important to ensure a robust parameterization?

## Materials and methods

### The grassland model GRASSMIND

The grassland model GRASSMIND is designed to simulate temperate grasslands and combines biogeochemical cycles with biodiversity, as well as plant-soil feedbacks, management and climatic effects [15]. The model follows the long tradition of forest gap models [23–27]. Individual plants interact and compete for resources on a rectangular patch (1 m x 1 m) without assignment of explicit spatial locations to each plant. Note that the gap approach for describing plant interactions (based on plant size differences) differs from spatially explicit approaches (e.g. of field-of-neighborhood type [28–30]) or more simplified approaches (e.g. mean-field approach [31, 32] or models that describe a species population by a representative plant [17,19,20,33,34]).

The model simulates the daily dynamics of each single plant within the community based on the following processes: (a) recruitment and emergence of plant seedlings, (b) plant senescence and mortality, (c) growth of plants (based on a carbon balance of photosynthesis and respiration), which can be (d) limited by environmental conditions or reduced due to interactions between plants. Intra- and interspecific competition for resources encompass aboveground ‘light’ and ‘space’ resources as well as belowground ‘soil water’ and ‘nitrogen’ resources. Limitations of growth can be caused, for example, by low light intensities (e.g. in winter or due to shading by other plants), unfavorable air temperatures, crowded space (‘thinning’), reduced soil water or soil nitrogen (e.g. in times of drought or due to strong competition with other plants).

Stochasticity is included in the model only in the mortality of plants. Plants can die due to an intrinsic mortality rate or in case when space is limited. While the intrinsic mortality acts at each day, mortality due to crowding is only triggered when growing plants and invading seedlings would cover more than the respective simulation area. Stochasticity is affecting which plant (of which size) is dying in each time step and thus, can have an impact on the simulated dynamics and structure of the grassland.

Simulated individual plants can differ in size (which is changing during the simulation) and species identity (which is defined by species traits fixed at the beginning of a plant’s life cycle). Some species traits affect size growth of single plants directly (e.g. leaf photosynthesis rate) while other traits rather affect plant interactions and community dynamics (e.g. seedling germination). By this, the model allows simulating monocultures as well as multi-species mixtures (large numbers of species can be simulated, but strongly increase runtime efforts). Concepts to simulate grasses, forbs and legumes typically occurring in temperate grasslands (e.g. in Europe) are included in the model. Soil water, carbon and nitrogen dynamics are modeled in this study using the Century soil model [35]. The GRASSMIND model allows to simulate large areas (e.g. hundreds of m^2^) and long-term grassland dynamics (e.g. decades). A detailed model description of GRASSMIND is provided in S1 Appendix.

### Study site and empirical data

For the parameterization of the grassland model, we used empirical data on a local biodiversity grassland experiment carried out in Central Germany (Jena Experiment, Germany, 50°55´N, 11°35´E, [7, 36], seeding year 2002). In this study, we firstly tested the GRASSMIND model and focused on two grass species (*Festuca pratensis*, *Poa pratensis*) and one forb (*Plantago lanceolata*) for which measurements on monoculture plots (20 m x 20 m) of each species and one two-species-mixture plot (of *P*. *pratensis* and *P*. *lanceolata*) are available. All plots were mown twice a year to a height of 10 cm (which is included in the simulation model). In the first year, plots were mown twice, eight weeks after sowing (in July) and in September [37] (11 July and 20 September). In all consecutive years (2003 to 2008), plots were mown twice a year in June and September [36] (20 June and 20 September). Plots have not been fertilized.

Plots have been measured biannually in terms of four vegetation attributes: aboveground biomass (AGB), leaf area index (LAI), vegetation height, and vegetation cover [36]. In the two-species-mixture plot, AGB and vegetation cover have been measured for each of the two species separately. In this study, we used published time-series data of observations from 2003 to 2008 [36].

**Fig 1:**
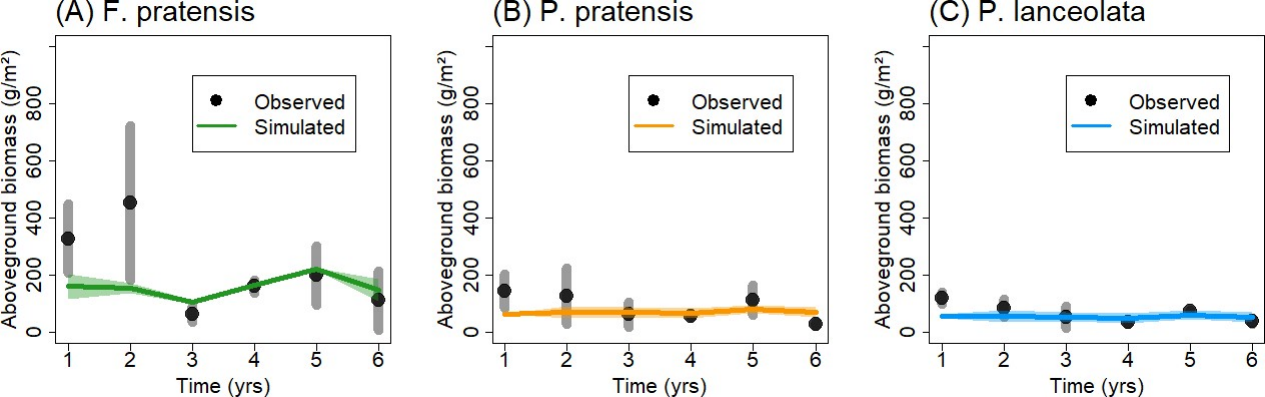
Aboveground biomass dynamics for three monocultures. Black dots show observed values and colored lines represent the simulated dynamics of (A) *F*. *pratensis*, (B) *P*. *pratensis* and (C) *P*. *lanceolata* using the model GRASSMIND (annual average and vertical grey lines/polygons denote the intra-annual range of two censuses per year).

Here, we aggregated biannual measurements to annual time-series data (mean value per year to reduce the variability in the observation, Fig 1, see S1 Fig for a comparison of using annual or bi-annual data in the model parameterization). Based on the calculated annual time-series from 2003 to 2008, we define the following ten ‘vegetation patterns’: time-series of (i) AGB, (ii) LAI, (iii) vegetation height and (iv) cover of each monoculture plot, time-series of (v) community LAI and (vi) vegetation height of the mixture plot, time-series of species-specific (vii) AGB and (viii) vegetation cover in the mixture plot, and time-series of relative yields (i.e. the ratio of an attribute value in the mixture to its value in the monoculture; in terms of (ix) AGB and (x) vegetation cover) per species. More details on the available empirical data can be found in S2 Appendix.

### Model parameterization

The GRASSMIND model was parameterized for three species (*Festuca pratensis*, *Poa pratensis*, *Plantago lanceolata*). As a result, we obtained species-specific sets of trait parameters which in combination with site-specific time-series of climate data, mowing dates and additional plot-based parameters (e.g. for soil attributes) were used to simulate the observed vegetation patterns in each field plot with the GRASSMIND model. To be able to compare daily simulated dynamics of individual plants with the observed vegetation attributes at the population and community level, we aggregated our simulation results. At the same biannual measurement dates of each field plot, we summed up simulated single plant biomass to calculate community level AGB (or only plants of a certain species for population level AGB; similarly calculated for vegetation cover). To obtain LAI, we summed up all single plant’s leaf area and related them to the simulated plot area. Vegetation height was determined as the height of the largest plant. The resulting simulation data of population and community level dynamics for each of the biannual measurement dates were then aggregated to annual time-series data (similar to the observations).

The model requires a total of 30 parameter values per species, from which nine parameters were available from literature (S2 Appendix). From the remaining parameters, five were estimated and the other 16 parameters (of plant geometry, physiology, demography and resource demand) parameterized inversely using optimization techniques [38, Tables 1 and 2]. To inversely determine parameter values, we used the observed vegetation patterns (see paragraph before, S2 Appendix). According to a selected algorithm, many parameter sets were constructed and tested in order to minimize the distances between observations and simulations (distances are thereby summarized in a cost function, S2 Fig, [38]).

**Table 1:** Geometrical parameters for the three species.

Parameter	Unit	Description	*F*. *pratensis*	*P*. *pratensis*	*P*. *lanceolata*	Reference
*h*_*max*_	cm	maximum height of a plant	120	60	70	[39–41]
*hw*	-	height-width ratio of a plant’s encasing cylinder	1.5	0.5	0.6	inversely parameterized
*f*_*s*_	g cm^-3^	shoot correction factor	0.00072	0.00057	0.00041	inversely parameterized
*f*_*O*_	-	overlapping factor	1	1	0.8	predefined (for *F*. *pratensis* and *P*. *pratensis*) inversely parameterized (for *P*. *lanceolata*)
*SLA*	cm^2^ g^-1^	specific leaf area	117.48	130.05	197.25	inversely parameterized
*SRL*	cm g^-1^	specific root length	42462.8	17804	89051. 1	inversely parameterized
*r*_*1*_,*r*_*2*_	-	parameters of the rooting depth power-law relationship	3.506 0.301	3.506 0.301	5.777 0.365	[42]
*sr*	-	shoot-root ratio of plant biomass organs	2.2	3.5	10.4	[37]

The table provides details about GRASSMIND parameters, with their units of measurements and prescribed or inversely parameterized values. While no overlapping of grass species was included (predefined factor of 1), we allowed plant overlapping for the forb species and calibrated its parameter inversely.

**Table 2:** Model process parameters for the three species.

Process	Parameter	Unit	Description	*F*. *pratensis*	*P*. *pratensis*	*P*. *lanceolata*	Reference
**Recruitment and emergence of new seedlings**	$N_{seed}^{meta}$	m^-2^ d^-1^	seed rain from surrounding landscape	4013	1062 (1934)	3038 (1100)	inversely parameterized
	*t*_meta_	julian day	julian day at which seed rain starts	136	136	136	[36]
	*t*_*em*_	d	time between seed rain and seedling emergence	14	21	7	[37]
	*germ*_*%*_	-	seed germination rate	0.3	0.75	0.9	[43]
	*h*_min_	m	initial height of ingrowing seedlings	0.03	0.03	0.03	predefined (technical parameter)
	*age*_rep_	yr	age at which recruitment starts	0.2	0.09	0.02	inversely parameterized
**Mortality**	*LLS*	d	leaf life span (start of yellowing leaves)	219	80	77	inversely parameterized
	*RLS*	d	root life span	166	278	547	inversely parameterized
	*m*_seed_	d^-1^	mortality rate of established seedlings	0.038	0.06	0.046	inversely parameterized
	*m*_basic_	yr^-1^	mortality rate of mature plants	0.02	0.02	0.02	[44]
	*life*	yr	life span of plants	perennial	perennial	perennial	predefined
**Photosynthesis**	*p*_*max*_	μmol_CO2_ m^-2^ s^-1^	maximum gross leaf photosynthesis	17	29	37	inversely parameterized
	*α*	μmol_CO2_ μmol_photons_^-1^	initial slope of light response curve	0.07	0.09	0.04	inversely parameterized
	*k*	-	light extinction coefficient	0.8	0.3	0.27	inversely parameterized
	*m*	-	transmission coefficient	0.1	0.1	0.1	[45]
**Competition**	*WUE*	g_ODM_ kg_H2O_^-1^	water use efficiency coefficient	2.7	6.1	7.5	inversely parameterized
	*CN*_green_	-	CN ratio of green leaves	18	27	23	inversely parameterized
	*CN*_sen_	-	CN ratio of senescent leaves and roots	39	47	49	inversely parameterized
	*N*_fix_	y/n	symbiotic N fixation	No	no	no	predefined
**Growth**	*alloc*_*shoot*_	-	allocation rate of *NPP* to shoot growth	0.50	0.33	0.35	inversely parameterized
	*r*_m_	d^-1^	maintenance respiration rate	0.02	0.02	0.02	[46]
	*r*_g_	-	growth respiration factor	0.2	0.2	0.2	[46]

The table provides details about GRASSMIND parameters, with their units of measurements and prescribed or inversely parameterized values. For seed rain of *P*. *pratensis* and *P*. *lanceolata* values of the monocultures are given first while mixture values are provided in brackets below.

The cost function for the optimization includes in our study all vegetation patterns of the annual time-series observations (aboveground biomass, leaf area index, vegetation height, vegetation cover of monocultures and species mixtures as well as their relative yields; see paragraph before for details). For a selected pattern *p* and species *i* we calculated the mean absolute percentage error (*MAPE*, [47]) of the simulated (*y_p,i_*) and observed (*x_p,i_*) time-series data. By this, the cost function includes (a) the growth behavior of three species in monocultures, (b) the growth behavior of a grass and forb species in a mixture and (c) the growth behavior of each species in a mixture compared to its monoculture for the grass-forb community (i.e. relative yields). Details, explanations and results of the model parameterization are described in the S2 Appendix.

In addition, we tested the inverse parameterization method for (i) different vegetation patterns included in the cost function, (ii) different algorithms for minimizing the cost function, (iii) different mathematical formulations of the cost function, and (iv) different degrees of detail of the observations included in the cost function. This sensitivity analysis is illustrated on the example of the monoculture plot of *Festuca pratensis* (due to extensive runtime efforts). Again, optimization was done using the previously introduced cost function (except for (iii)) and evaluated using our model evaluation criteria (introduced in the next subsection).

For (i) we tested whether fitting only one single vegetation pattern could be sufficient to explain population dynamics rather than including multiple patterns. In (ii) we tested different cost functions on the example of aboveground biomass: mean absolute percentage error (*MAPE*), normalized root mean square error (*nrmse*) and a combined function based on our evaluation criteria (for details see S2 Appendix). For (iii) we analyzed three different algorithms on the example of aboveground biomass: dynamic dimensional search (DDS), adaptive simulated annealing (ASA) and differential evolution (DE). Details on the algorithms can be found in [38]. In (iv) we compared the optimization if we include annual or biannual time-series in the cost function on the example of leaf area index (as this represents a well calibrated attribute in contrast to AGB).

### Model evaluation

To evaluate the accuracy of our model parameterization, we compared the annual time-series of observations and simulations for each vegetation pattern (similarly aggregated as for the model parameterization; average of 100 replicated model simulations to account for stochasticity, Table 1). For descriptive purposes only, we also averaged values of each observed and simulated vegetation pattern (to calculate an average value of annual mean and standard deviation across years 2003 to 2008; sample size N = 6 years; Table 1).

For a selected pattern *p* and species *i* we performed a linear regression of the simulated (*y_p,i_*) and observed (*x_p,i_*) time-series data: *y_p,i_* = *I_p,i_*+*s_p,i_*⋅*x_p,i_* (resulting in a slope *s_p,i_*, intercept *I_p,i_* and the coefficient of determination *R*^2^, sample size N = (6 years × 3 species) for the monocultures, N = 6 years for the mixture). In addition, we calculated the normalized root mean square error *nrmse* (sample size N = (6 years × 3 species) for the monocultures, N = 6 years for the mixture).

**Fig 2:**
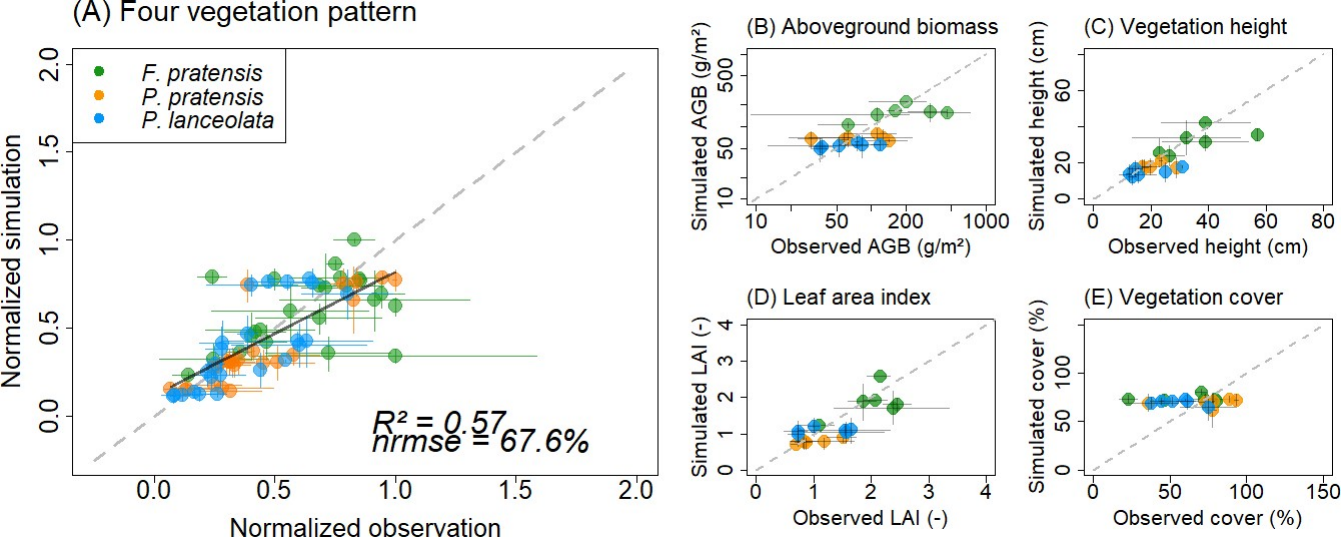
Comparison of observed and simulated patterns in terms of aboveground biomass, vegetation height, leaf area index and vegetation cover for three monocultures. Each dot reflects a yearly value. Colors mark results for the different species. In (A) all compared patterns for the three species are combined. Different patterns are normalized by the maximum value (of observations and simulations for all species). The black solid line shows the linear regression line for which the *R*^2^ and *nrmse* is displayed. In (B-E) each pattern is displayed without normalization. Note that axes for AGB are logarithmic.

**Fig 3:**
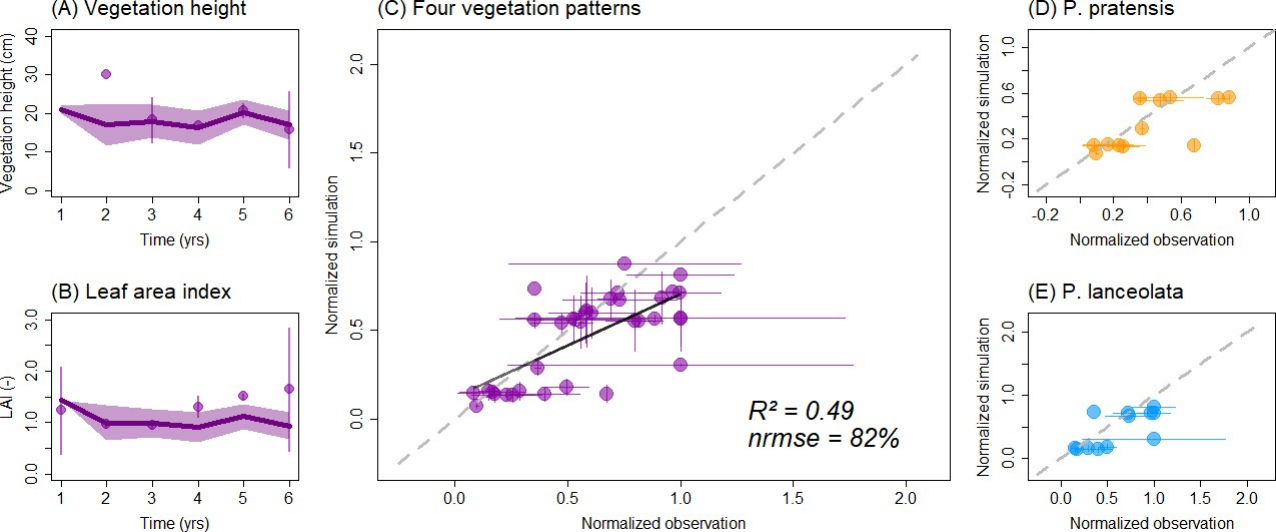
Comparison of observed and simulated patterns for the two-species-mixture. In (A-B) annual dynamics of AGB and LAI are shown. In (C-E) each dot represents an annual value compared between observation and simulation. In (A, D-E) species are marked by different colors (yellow and blue). In (C) all results are combined and normalized by the maximum value (of observations and simulations per selected pattern). In (D) the observed AGB and vegetation cover is compared with the simulated values only for *P*. *pratensis* within the mixture, while in (E) both patterns are shown only for *P*. *lanceolata*’s contribution in the mixture (again normalized by their respective maximum values).

To get an overall impression, we further compared and analyzed the normalized observed and simulated vegetation patterns of (a) all monoculture plots (Fig 2A) and (b) all attributes in the mixture plot (Fig 3C). For this overall evaluation, we divided simulated and observed vegetation patterns by the maximum value of observed and simulated data of all species, but per pattern separately. Details, explanations and results of the model evaluation are described in the S2 Appendix and S3 Fig.

## Results

### Species population dynamics in monocultures

The observed population dynamics in monocultures (two grasses and one forb species) show considerable differences in their intra- and inter-annual variations (Fig 1, Table 3, S4 Fig). Aboveground biomass (AGB) ranges on average between 44 to 328 g per m^2^; especially *F*. *pratensis* shows peaks during the first years of transient growth phase with an average biomass of 450 g per m^2^ in 2004. Species-specific dynamics of leaf area index (LAI) and vegetation height follow nearly similar annual patterns compared to aboveground biomass (S4 Fig). Both grass species differ considerably as *P*. *pratensis* reaches only about half of the biomass (AGB), LAI and vegetation height of *F*. *pratensis* (S4 Fig, Table 3).

**Table 3:** Comparison of observed and simulated vegetation patterns and its evaluation.

Plot	Pattern	Species	Observed	Simulated	*MAPE*	*Regression slope* (*R*^2^)	*nrmse* (%)
**Mono-cultures**	AGB	*F*. *pratensis*	219.6 (111.8–327.5)	158.7 (139.8–177.5)	0.38	0.31 (0.44)	80.3%
		*P*. *pratensis*	88.8 (44.3–133.4)	70.2 (55.9–84.4)	0.48		
		*P*. *lanceolata*	67.4 (49.3–85.5)	55.4 (40.0–70.7)	0.32		
	LAI	*F*. *pratensis*	2.0 (1.6–2.4)	1.9 (1.6–2.1)	0.16	0.70 (0.64)	63.5%
		*P*. *pratensis*	1.0 (0.8–1.2)	0.8 (0.6–1.0)	0.16		
		*P*. *lanceolata*	1.2 (0.9–1.6)	1.1 (0.8–1.4)	033		
	Height	*F*. *pratensis*	36.1 (27.1–45.2)	32.2 (27.0–37.4)	0.15	0.61 (0.67)	63.2%
		*P*. *pratensis*	20.2 (18.8–21.5)	17.8 (14.2–21.5)	0.12		
		*P*. *lanceolata*	18.7 (16.8–20.6)	15.0 (11.0–19.0)	0.22		
	Cover	*F*. *pratensis*	61.8 (56.1–67.5)	74.1 (70.7–77.6)	0.53	-0.01 (0.002)	106.9%
		*P*. *pratensis*	74.5 (72.7–76.3)	69.7 (61.1–78.4)	0.28		
		*P*. *lanceolata*	55.0 (45.1–64.9)	70.1 (64.2–76.0)	0.39		
**Mixture**	AGB	*P*. *pratensis + P*. *lanceolata*	141.7 (86.7–196.8)	65.7 (50.5–80.9)	0.91	0.15 (0.62)	105.2%
	LAI		1.3 (0.9–1.7)	1.0 (0.8–1.3)	0.20	-0.02 (0.0006)	134%
	Height		20.3 (16.8–23.8)	18.2 (14.6–21.9)	0.12	0.01 (0.003)	101.9%
	Cover		77.2 (68.4–86.0)	69.9 (63.0–76.9)	0.59	0.30 (0.31)	83.5%

Average values (years 2003 to 2008) of yearly mean (in brackets the average of yearly minimum and maximum values is given) in terms of aboveground biomass (AGB), leaf area index (LAI), vegetation height and cover are compared for three monocultures and a two-species community.

**Fig 4:**
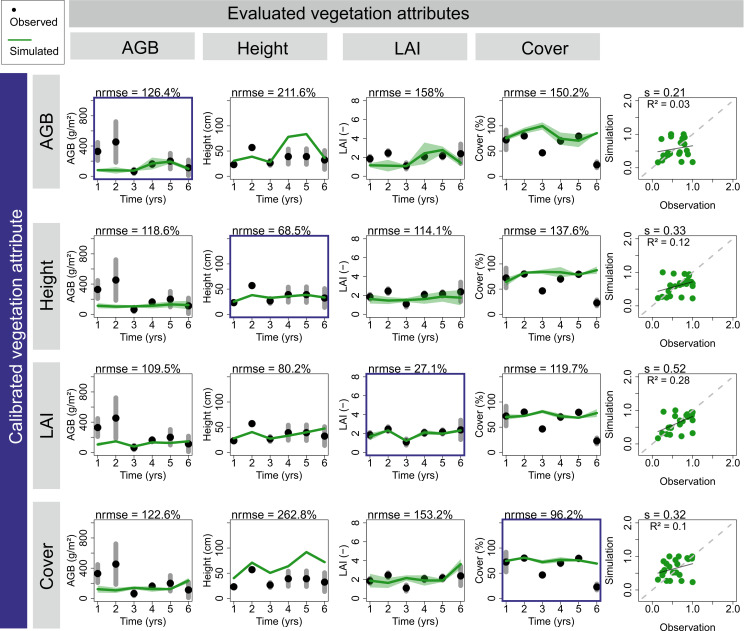
Comparison of different observed vegetation attributes included as single attribute in the inverse model parameterization. The calibrated vegetation pattern is framed by a blue rectangle while the other vegetation patterns are shown for evaluation purposes (example of *F*. *pratensis* monoculture, using *MAPE* as cost function). Green lines (and shaded polygons) describe simulations (yearly mean and range) while black dots and grey lines describe the observations (yearly mean and range). All four vegetation patterns are normalized and summarized in a 1:1 plot (right panel). See methods for details.

Monoculture simulations match only partly the observed vegetation patterns with an overall moderate *R*^2^ of 0.57 and *nrmse* of 67.6% (regression slope *s* = 0.7; Fig 2, Table 3, S4 Fig, S2 Appendix). Some deviations remain unresolved, mostly with regard to aboveground biomass and vegetation cover of *F*. *pratensis* (S4 Fig, S2 Appendix). The dynamics of LAI and vegetation height show comparably low *nrmse* values for all species (Fig 4B–4E, Table 3). The observed inter-annual range of LAI, vegetation height and cover can be reproduced by the simulation model, while for AGB the observed range was on average two times higher than simulated (Table 3).

### Dynamics and interactions in two-species mixtures

Simulated patterns of grassland dynamics for both species in the mixture show large deviations to those observed in the field with an overall high *nrmse* of 82% and low *R*^2^ of 0.49 (regression slope *s* = 0.58; Table 3, Fig 3, S5 and S6 Figs). Among all patterns, vegetation cover was the best reproduced pattern by the model (*nrmse* < 100%; Table 3, S2 Appendix) compared to vegetation height, AGB and LAI (*nrmse* > 100%; Table 3). The dynamics of species composition is simulated in sufficient agreement with empirical measurements (for cover and AGB; Fig 3D–3E, S5 and S6 Figs). Observed inter-annual ranges were well reproduced by the simulations (Table 3), except for AGB which was about factor two to three lower in the simulations.

When comparing the observed behavior of monocultures (for *P*. *pratensis* and *P*. *lanceolata*) with their respective outcome in the mixture, we notice that the forb species (*P*. *lanceolata*) is more competitive in terms of AGB compared to the grass species (*P*. *pratensis*). While the forb is able to exceed its expectations (i.e. half of its monoculture), the grass species is observed far below the forb and reaches, for example in year 2003 only around 35% of its expectations (S7 Fig, S2 Appendix). Most of the simulated values of relative yields in terms of cover coincide with the observed values, while simulated relative yields in terms of AGB (especially for *P*. *lanecolata*) fail to match the observations (S2 Appendix, S7 Fig).

### Insights from model parameterization

Best matches between observations and simulations were obtained when accounting for all available information on the different vegetation patterns (‘multi-constrained inverse parameterization’). In a demonstration example of an *F*. *pratensis* monoculture, calibrating time-series only on single vegetation patterns optimizes the simulation of the respective pattern (*nrmse* values of 27% - 126%), but clearly fails when comparing the excluded attributes (Fig 4, *nrmse* values between 80% and 263%). Moreover, for aboveground biomass even the calibrated pattern was not reproduced well (Fig 4, *nrmse* ≈ 126%).

Interestingly, calibrating LAI alone already provides comparably sufficient matches of vegetation height and AGB (*nrmse* ≈ 80% - 110%) but less for cover (*nrmse* ≈ 127%). This result can also be assessed when combining multiple patterns for the calibration (e.g. two patterns, three patterns or full information). Adding additional information to the calibration improves clearly those patterns not calibrated before, but often at the expense of those patterns previously included in the calibration (S8 and S9 Figs). In all possible combinations of observed vegetation patterns included in the calibration, the inclusion of LAI revealed in most cases best results (comparably low *nrmse* in all patterns). Nevertheless, using all available information still showed the best calibration result. Additional effects can be expected when using alternative calibration algorithms or cost functions (S10 and S11 Figs).

## Discussion

Here we tested in a first step the individual- and process-based grassland model GRASSMIND for simulating monocultures and a two-species mixture of two grass and one forb species (based on a mown grassland experiment in Central Germany). Key characteristics of the model include: (i) the ability to track the plant size structure in grasslands and by this, intra- and interspecific plant interactions, (ii) the calculation of plant growth based on biogeochemical cycles (in terms of carbon, nitrogen and water), (iii) the description of plant interactions by species traits and plant size, and (iv) the detailed modelling of soil and hydrological processes by coupling soil models. By this, simulated monocultures as well as species assembly in mixtures can reproduce general trends of empirical measurements for different vegetation attributes, however still with some considerably quantitative deviations. Parameterization of the model is partly challenging due to scarce plant measurements at the individual plant level, which can be overcome by inverse parameterization methods. We demonstrated that multiple vegetation attributes are important for a comprehensive model parameterization while leaf area index demonstrated to be of highest relevance for a robust parameterization. Failures in exactly matching all observation data at the same time pinpoint to the need of detailed grassland measurements (e.g. of higher temporal resolution) for advancing modelling approaches.

Extending the model’s applicability to other regions than the here analyzed study site requires additional tests of the GRASSMIND model on its transferability to various environmental conditions, management regimes (including model extensions to simulate grazing) and a higher richness of plant species and functional groups (including legumes). Accounting for additional factors and their integration in grassland models, however, needs careful consideration in terms of potential changes in model behavior [48], especially when moving to other ecoregions (e.g. Mediterranean grasslands characterized by shifted growing-seasons to autumn-spring, summer drought and variable precipitation [49]).

While the GRASSMIND model simulates the biogeochemical growth of individual plants interacting with each other in their neighborhood, other comparable grassland models simplify specific processes in favor of applicability [15]. Individual-centered models (e.g. GEMINI [19,33,34], or LPJmL [20]) simulate, for example, average plants per species within mixtures, but is at the expense of a detailed size structure of plants within grasslands. The mechanistic model IBC-grass [18,21] in turn is able to track the size of individual plants, but describes plant development by growth functions which exclude biogeochemical cycles. In another direction, an increasing model complexity can complicate model parameterization for species-rich mixtures and is thus often applied only for a representative mean species (e.g. Hurley Pasture Model [50]). Nevertheless, individual-based and individual-centered models enable to analyze morphological and physiological trait plasticity [19,33,34].

Mechanistic models support the understanding of complex processes and interactions in grasslands, whereas applications at larger scales benefit from simpler mechanistic or even empirical models [51]. Less complex model types (e.g. the generic CoSMo model [52,53], being a model component susceptible to be coupled to generic simulation models that deal with grasslands as an average plant species) are therefore often based only on specific vegetation attributes for simulating community changes and species composition (e.g. aboveground biomass, [53]). However, recently developed methodological frameworks for applying (or upscaling) process-based models to larger scales without losing multiple small-scale information have already been tested for various biomes (e.g. [54–57]) and can be promising to be transferred to temperate grasslands.

As climate change research becomes more and more relevant, grassland models can play a major role for understanding the interplay between environmental change, biodiversity and grassland functioning [58]. Next generation grassland models thereby are posed to specific challenges, including among others the improved representation of multi-species grasslands (e.g. for identifying important species traits and processes to enhance grassland resilience) as well as GHG balances, and promoting regional transferability of models to various environmental conditions [51].

### Challenges in confronting the grassland model with empirical data

Parameterizing the grassland model for the three selected grassland species included some challenges as some field observations were not able to be matched by the model (mostly in terms of aboveground biomass). Here, we were able to identify that the parameterization of an individual- and process-based grassland model like GRASSMIND requires the inclusion of several combined observed vegetation attributes (‘multi-constrained inverse parameterization’). To progress future model development and improve parameterization, further analysis of more computer-intensive techniques (like Approximate Baysian Computation [59,60], and related cross-validation procedures [61]) can provide additional insights on the uncertainty of estimated model parameters.

Dependent on the location of observation plots, rates of seed rain as well as the functional type of undesired species or weeds in the biodiversity experiment can differ. Such differences can cause variations in species dynamics and interactions on the plot (e.g. by species invaders or weeds), which could also propagate to the measured vegetation patterns (e.g. aboveground biomass) causing the observed variations. Although biodiversity experiments have the advantage to stepwise confront models with population and community dynamics of various species richness, natural monitoring sites (e.g. Nutrient Network sites, [5]), which allow ingrowth and persistence of all species of the regional species pool, could overcome these uncertainties.

Empirical time-series data of grasslands often lack continuous measurements. Various vegetation patterns at a high temporal resolution have so far been rarely measured over longer time periods (e.g. monthly or weekly measurements over several years). Although the process-based grassland model simulates on a daily basis, we thus can only compare specific points in time within a year (here in this study, two days per year). By this, some model parameters (e.g. plant architecture) which still remain difficult to measure are also challenging during inverse model parameterization. In addition, plant attributes are constant species trait parameters in the model, but may vary in reality over the lifetime of an individual plant (e.g. *specific leaf area* [62]). Model development in the direction of trait evolution or adaptation to environmental conditions could potentially integrate such effects.

Multiple patterns of empirical measurements are in general desired to sufficiently parameterize individual- and process-based grassland models [63]. The here applied parameterization methods in fact required multiple patterns which may reflect different structural and physiological attributes of grasslands. Data on LAI and vegetation height, for example, describe the vertical size structure of grasslands in an aggregated way, thereby pinpointing to potential plant size hierarchies within the community. In turn, vegetation cover reflects rather a measure of lateral spread of species and thus, of horizontal vegetation structure (or implicit spatial structures). Aboveground biomass is an additional component for integrating physiological processes correctly which drive grassland productivity and plant density (correlating with leaf area index).

The inclusion of multiple vegetation patterns describing the vertical and horizontal structures as well as physiological aspects thus affect the parameterization of different species traits for the presented grassland model. While attributes of a species’ light response mostly affect aboveground plant biomass, geometric species traits (e.g. plant shoot architecture) influence vegetation height and cover much stronger. Nevertheless, all species traits interfere in the model via intra- and inter-specific interaction dynamics and interplay strongly with plant size hierarchy. An open question remains if (and which) observed vegetation attributes are sufficient for model parameterization in general (i.e. beyond the selected plots tested in this study). Here, we highlighted the relevance of LAI, thus calibrating for example only LAI time-series, could be a promising approach for IBM-parameterizations of grasslands. Leaf area index can generally be provided in a high spatial and temporal resolution (e.g. daily) by remote sensing while ground-truth measurements (e.g. on aboveground biomass and vegetation height) could be used for validation then.

### Perspectives of grassland modelling

Grasslands have gained large attention during the last decades by the set-up of biodiversity experiments and global monitoring campaigns, which relate species diversity to ecosystem functioning at the local scale as well as across spatial scales. Empirical analyses of such studies already highlighted for example, that species yield can differ in mixtures compared to monocultures. Empirically observed diversity-productivity relationships have revealed that positive effects are mainly driven by an increasing plant density of overyielding species (predominantly forbs and legumes, [64]).

To understand why species in mixtures differ in their competitive dynamics compared to monocultures, grassland modelling is valuable in complementing field measurements. Insights have been gained, for example, by structural equation modelling [65] and simple mechanistic models [11, 66, 67]. In contrast, process-based models allow to look deeper into mechanisms and enable to switch on or off different processes (e.g. competition for water between plants), to manipulate specific site conditions (e.g. soil properties), to derive additional attributes and to upscale local measurements. By this, the effect and strength of ecosystem processes and external drivers on grassland dynamics, structure and species interactions can be analyzed and disentangled in a systematic manner. This approach complements field experiments which are often limited in the number of factors that can be varied within their designs. Efforts for long-term monitoring and analysis of experiments increase with variations of species’ seed numbers, soil properties and management (including the prevention of invaders) combined with a full design of a field experiment in terms of species diversity and assembly.

Accounting for heterogeneities in the environment for grassland dynamics especially becomes relevant when analyzing study sites at larger spatial scales or comparing various sites across spatial scales. Still, the ongoing debate about generalized diversity-productivity relations at local sites and productivity-richness relations across scales, as well as their linkage opens up new research questions and hypotheses [5, 9, 10, 68]. Applying a process-based model to larger spatial scales enables to analyze the interplay of species dynamics and ecosystem function (e.g. productivity or soil carbon storage) for local and regional scales (e.g. in virtual landscapes of heterogeneous soil, climate, anthropogenic management and natural disturbances). Besides spatial heterogeneities, also temporal variability in the environment (e.g. rainfall duration and intensity) and in natural or anthropogenic disturbances (e.g. stochastic extreme events or regular mowing) needs to be considered to understand the stability and recovery of grasslands, especially in terms of functioning and species coexistence [69–73]. Analyses, for example, on how long environmental change or disturbances impact grassland ecosystems can only be supported when field studies run on the long-term. Species traits have strongly been in the focus of biodiversity-ecosystem-functioning studies with the general hope to infer long-term ecosystem properties only from the prevailing species traits [66]. The set-up of large trait databases like TRY [74], e-FLORA [75] or other initiatives (e.g. Glopnet [76] or LEDA [77]) supported such analysis and stimulated modelling efforts using species trait distributions [78,79]. Models which integrate trait distribution concepts, for example, randomly choose a few independent traits for new recruits while all others are derived by trade-off relations. Another approach encompasses to completely dice out all species traits without predefining trade-offs or trait ranges [80–82]. Process-based models can be used as a filter to derive species trait distributions and trade-offs restricted to match specific criteria of ecosystems (e.g. ecosystem structure, long-term species coexistence and stability). Such pattern-oriented filtering further can help to infer underlying mechanisms not only from prevailing species traits, but also from attributes recorded in monitoring campaigns at various spatial and temporal scales [83, 84]. Species traits collected in large databases can substantiate model parameterization of species-rich communities and can help to identify correlations between species traits. However, sample size and location of collected traits are still not well-balanced across regions [85].

Satellite-based remote sensing provides novel approaches to long-term monitor grasslands in high-resolution, but also still lack detailed knowledge beyond NDVI measurements (e.g. MODIS products, [86]), the extent of grassland cover (e.g. [87–91]) and diversity indices (e.g. [92]). In contrast, for forest ecosystems remote sensing already provides detailed knowledge on forest states, for example in terms of forest structure and also partly species trait composition [93–95]. Studies that simulate remote sensing measurements within process-based forest models (successfully applied for managed temperate and species-rich tropical forests) additionally aided the reliable interpretation of remotely sensed signals and derived indices [96,97]. Such combination of process-based grassland modelling with remote sensing measurements could also open up new perspectives for interpreting remote sensing observations and extracting knowledge on the state and dynamics of grasslands.

## Supporting information

S1 Appendix GRASSMIND 2.0 –grassland model. (PDF)

S2 Appendix Additional information on empirical data and input parameter of the grassland model GRASSMIND. (PDF)

S1 Fig Comparison of different degrees of detail of observation data on LAI used for the inverse model parameterization. The example of *F*. *pratensis* monoculture is shown here. The calibrated vegetation pattern (yearly mean values versus bi-annual measurements) is framed by a blue rectangle (for bi-annual shown as inlet) while the other vegetation patterns are shown for evaluation purposes. All four vegetation patterns are normalized and summarized in a 1:1 plot (right panel).
(TIF)

S2 Fig Illustration of the inverse model parameterization. In (A) each optimization and the corresponding cost function is shown (using *MAPE* as cost function and the *DDS* algorithm for optimization). In (B) only successfully minimized optimization steps are displayed. See methods for details.
(TIF)

S3 Fig Illustration of the evaluation criteria. Examples of comparisons between virtually created observed and simulated data points (black dots) to illustrate the different evaluation criteria (the linear regression line is visualized in blue). In (A) virtual simulations match perfectly virtual observations. In (B) the qualitative trend of observations is reproduced perfectly by simulations (indicated by slope *s* = 1 and *R*^2^ of 1) while the positive intercept and *nrmse* shows a systematic deviation. In (C) simulations and observations deviate from each other (reflected by the low *R*^2^ and high *nrmse*) but reproduce on average the qualitative and quantitative trends (due to intercept of *I* = 0 and slope of *s* = 1). In (D) and (E) examples which do not fulfill any criteria optimally are shown. In each panel, the 1:1 grey dotted line illustrates optimal evaluation criteria.
(TIF)

S4 Fig Dynamics of vegetation height, LAI and vegetation cover for three species monocultures. Black dots show observed values and colored lines represent the simulated dynamics using GRASSMIND (black dots/colored lines show the annual average and vertical grey lines/polygons denote the range of two single measurement values per year).
(TIF)

S5 Fig Comparison of observed and simulated patterns in terms of aboveground biomass (AGB), vegetation height and cover and leaf area index (LAI) for the species mixture. Each dot reflects the comparison of an observed with a simulated yearly value of a selected pattern. Colors identify the two species (orange–*P*. *pratensis*, blue–*P*. *lanceolata*, purple–total community).
(TIF)

S6 Fig Dynamics of (A) aboveground biomass and (B) vegetation cover for the species mixture. Dots show observed values and colored lines represent the simulated dynamics using GRASSMIND (both show the annual average and vertical grey lines/polygons denote the range of two census values per year).
(TIF)

S7 Fig Relative yield of (A) vegetation cover and (B) aboveground biomass for two species included in the mixture. Bars show the simulated mixture performance of a species divided by its monoculture performance. Black dots show the analogous empirical data. A relative yield of 1 (green dotted line) means that the species behaves in the mixture similar compared to its monoculture (although in competition for space and resources with the other species). The red dotted line (value of 0.5) represents the expected relative yield of a species in the mixture (assuming both species equally distribute resources among them).
(TIF)

S8 Fig Comparison of different observed vegetation attributes included as two-pair combination in the inverse model parameterization. The calibrated vegetation patterns are framed by a blue rectangle while the other vegetation patterns are shown for evaluation purposes (example of *F*. *pratensis* monoculture, using *MAPE* as cost function). Green lines (and shaded polygons) describe simulations (yearly mean and range) while black dots and grey lines describe the observations (yearly mean and range). All four vegetation patterns are normalized and summarized in a 1:1 plot (right panel).
(TIF)

S9 Fig Comparison of different observed vegetation attributes included as three-pair and four-pair combinations in the inverse model parameterization. The calibrated vegetation patterns are framed by a blue rectangle while the other vegetation patterns are shown for evaluation purposes (example of *F*. *pratensis* monoculture, using *MAPE* as cost function). Green lines (and shaded polygons) describe simulations (yearly mean and range) while black dots and grey lines describe the observations (yearly mean and range). All four vegetation patterns are normalized and summarized in a 1:1 plot (right panel).
(TIF)

S10 Fig Comparison of different calibration methods for the inverse model parameterization (example of *F*. *pratensis* monoculture, using *MAPE* as cost function). The calibrated vegetation pattern (here on the example of AGB) is framed by a blue rectangle while the other vegetation patterns are shown for evaluation purposes. Green lines (and shaded polygons) describe simulations and black dots (with grey lines) the observations (yearly mean and annual range). All four vegetation patterns are normalized and summarized in a 1:1 plot (right panel).
(TIF)

S11 Fig Comparison of different cost functions for the inverse model parameterization (example of *F*. *pratensis* monoculture). The calibrated vegetation pattern (here on the example of AGB) is framed by a blue rectangle while the other vegetation patterns are shown for evaluation purposes. Green lines (and shaded polygons) describe simulations and black dots (with grey lines) the observations (yearly average and annual range). All four vegetation patterns are normalized and summarized in a 1:1 plot (right panel).
(TIF)
