# Supplementary material for: Confronting an individual-based simulation model with empirical community patterns of grasslands
Source: PLoS One. 2020 Jul 28;15(7):e0236546. doi: 10.1371/journal.pone.0236546 (PMC7386574; doi:10.1371/journal.pone.0236546)
Supplement: S1 Appendix — (PDF) [file pone.0236546.s001.pdf]

S1 Appendix

GRASSMIND 2.0 – grassland model

1 Overview and general concept

The grassland model GRASSMIND is designed to simulate grasslands and combines biogeochemical cycles with biodiversity [1]. The model includes additional submodels that allow for analyzing plant-soil feedbacks and effects of management and climate change. A first conceptual model description (GRASSMIND 1.0) has been described earlier [1]. Since then, progress in the model development has been made in terms of modelling the nitrogen demand of plants and nitrogen relocation, as well as the coupling with soil models.

GRASSMIND 2.0 is an **individual-based and process-oriented** model that follows ~~the~~ a gap approach typically applied in forest models [2-5]. Grassland is simulated on an area of size A (m^2), which is a composite of regularly ordered, quadratic patches of a = 1 m^2 in size and described by their location within the area A (S1.1 Fig). Individual plants interact and compete for resources on one patch without assignment of explicit spatial locations to each plant (S1.1 Fig). Intra- and interspecific competition for resources and their resulting uptake influence the productivity of plants. Aboveground resources include light and space and belowground resources comprise soil water and nitrogen. Resource supply is assumed to be homogenous within each patch, but can differ between patches.

For simulating **soil resource dynamics**, GRASSMIND is coupled with two soil models: (i) CANDY [6] and (ii) CENTURY [7]. This study uses the soil model CENTURY. The monthly time step of CENTURY is matched with daily dynamics of GRASSMIND by updating soil water processes and carbon-nitrogen decomposition at the beginning of each month (while accumulating daily soil water uptake by plants and daily litter fall of plant leaves and roots).

Biogeochemical cycles include the carbon, nitrogen and water fluxes in the grassland ecosystem. The growth of single plants is modeled based on a carbon balance of gross primary production (*GPP*) and respiration. Adding leaf and root turnover and demographic process (like seed ingrowth and plant mortality), the carbon cycle is extended to the plant community and is closed via litter decomposition and soil carbon processes for the grassland ecosystem. The nitrogen cycle is modeled in close connection to the carbon cycle by assuming *CN* ratios for plants (green and senescent leaves and roots) and soil pools. The water cycle includes as main processes interception, evaporation, water infiltration and percolation in soil as well as plant transpiration.

Biodiversity is integrated in the model by either simulating single species or plant functional types (aggregated species with similar functional behavior; PFT). Species or PFT can differ in traits which determine their demographic rates, growth and behavior in competition with other plants. Plants of the same species or PFT do not differ in their traits, but can have different ages or sizes. The geometry of an individual plant is described by the aboveground shoot (here, leaves) and belowground root system (root branches).

The **processes** included in the grassland model are modeled according to a specific schedule (S1.1 Fig):
(A) recruitment and emergence of plant seedlings (1,2)
(B) plant senescence and mortality (3-5)
(C) gross production (incl. shading and competition for soil water and nitrogen, 6-10,13)
(D) plant respiration (for maintenance and growth, 11)
(E) net production and allocation for plant growth (12,14)
(F) management (15)

The grassland model runs at daily time steps (Δt = 1). For the purpose of shading and crowding mortality, the aboveground space is discretized into vertical height layers of constant width Δh (S1.1 Table).

Table S1.1. Overview of general input parameter for GRASSMIND.

Description	Parameter	Unit	Value
Time step	Δt	d	1
Simulation area	A	m^2	1 … 100
Patch area	a	m^2	1
Width of height layers	Δh	m	0.01

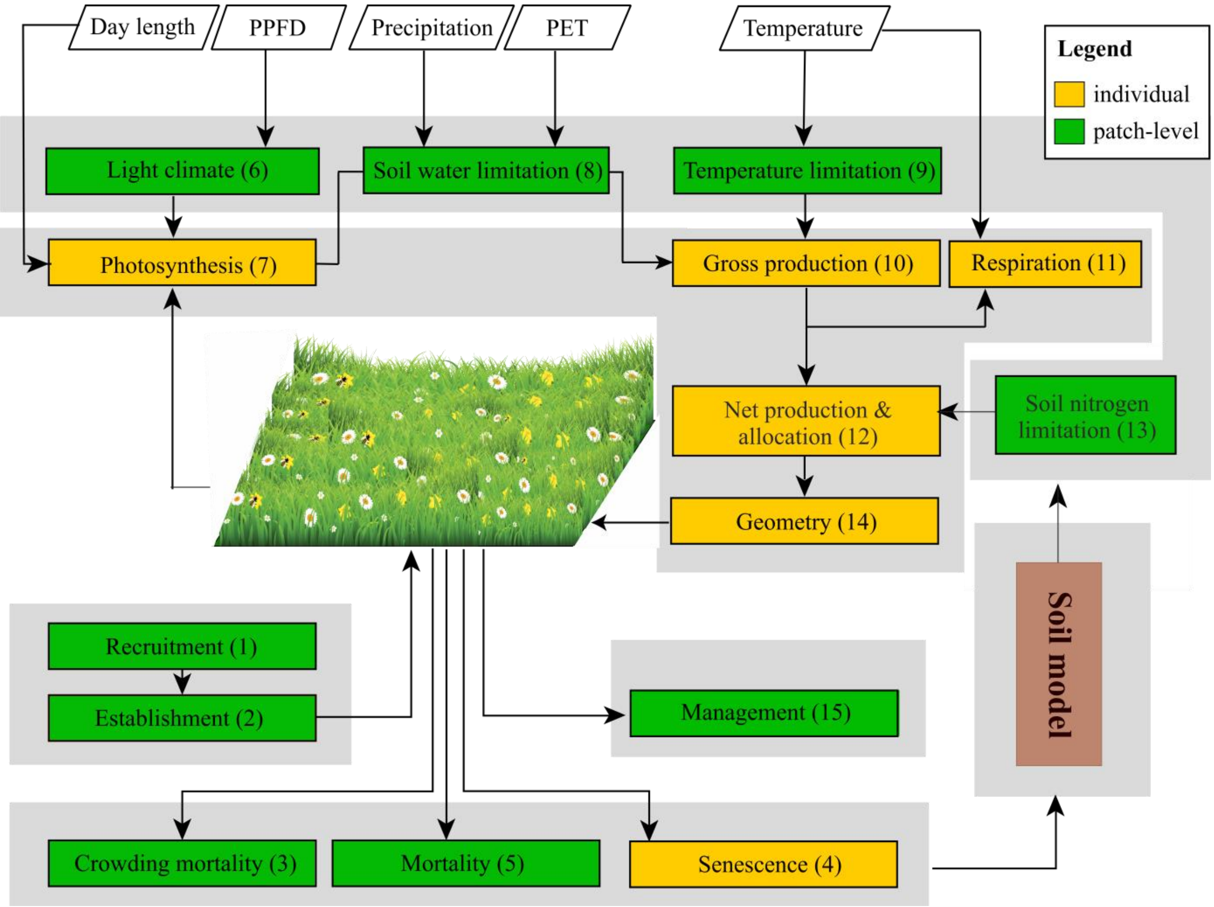

Fig S1.1. **Overview of main processes in GRASSMIND.**

Numbers in brackets within each box show the serial order of their calculation within one time step Δt. Grey frames that underlie these boxes group them according to the main processes and their corresponding chapters. Rhombuses indicate climatic input parameters with the following abbreviations: *PET* – potential evapotranspiration, *PPFD* – photoactive photon flux density. Spatial scale of a process is marked by different colors (green = patch, yellow = individual plant).

2 The geometry of an individual

Each individual plant is characterized by the following state variables (organic dry matter in g_{ODM}):

(1) aboveground shoot biomass B_{shoot}

(2) belowground root biomass B_{root}

(3) reproduction biomass B_{rep}

The aboveground shoot biomass is divided into biomass of fresh green leaves B_{shoot}^{green} and biomass of senescent yellow leaves B_{shoot}^{sen}. Further state variables, which describe the

geometry of an individual, can be derived from allometric relationships with species-specific attributes (S1.2 Fig).

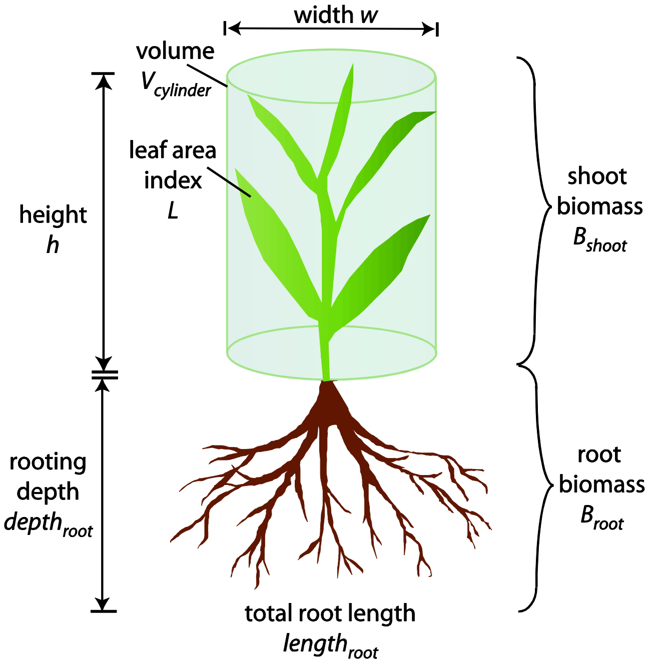

Fig S1.2. Geometry of a single plant in GRASSMIND.

State variables that correspond with the geometrical characteristics of an individual plant and that can be derived from the aboveground shoot and belowground root biomass.

2.1 The aboveground shoot

We model the aboveground shoot of an individual plant encased by a cylinder. The volume of the encasing cylinder $V_{cylinder}$ [m³] is related to the shoot biomass:

$$V_{cylinder} = \frac{B_{shoot}}{f_s} \tag{1}$$

where the species-specific correction factor f_s [g_{ODM}/m^3] accounts for free space within the cylinder not filled with biomass. A species-specific constant parameter *hw* defines the ratio between plant height *h* [m] and width *w* [m] of the assumed cylinder.

The space an individual plant occupies on a patch is determined by the ground area *cov* [m²] of the encasing cylinder. To calculate a patch's *vegetation cover CC* (the area occupied by all individuals relative to the patch area), it is necessary to take leaf overtopping or overlapping among individual plants into account. For this, each plant cover is corrected by a species-specific *overlapping factor* f_o (unitless). This factor accounts for overlapping in an implicit

manner since the individuals do not have spatially explicit positions within the patch. The corrected covers of all plants on the patch are summed up and normalized by the patch area:

$$CC = \frac{1}{a} \cdot \sum_{all\ individuals} (cov \cdot f_o) \tag{2}$$

The leaf area of the aboveground shoot is obtained by multiplying the plant's biomass B_{shoot} with the constant parameter of *specific leaf area SLA* [m^2/g_{ODM}]. This includes the assumption that all leaves have the same *SLA*, leading to the overall plant leaf area index L [m^2/m^2]:

$$L = \frac{B_{shoot} \cdot SLA}{cov} \tag{3}$$

By using only the green shoot biomass B_{shoot}^{green} instead of B_{shoot} in equation (3), we obtain the green leaf area index L_{green} which is especially important for photosynthesis (see section 3.5).

2.2 The belowground root

We assume a species-specific allometric relationship between an individual's aboveground shoot biomass B_{shoot} and belowground root biomass B_{root} – determined by the parameters *sr* (defining the species-specific *shoot-root ratio* of a plant in terms of biomass):

$$B_{shoot} = sr \cdot B_{root} \tag{4}$$

The individual's ability to access and compete for soil nitrogen and water resources strongly depends on its root system. In addition to root biomass, the root system's vertical distribution in soil is also considered. Shallow and highly branched root systems are beneficial for nutrient uptake as most nutrients occur predominantly in the upper soil layers. In contrast, deeper root systems strongly increase the individual's access to soil water resources, particularly during drought periods.

To calculate the rooting depth $depth_{root}$ [m], which is required for water uptake, we adapt the power-law approach [8] which functionally relates rooting depths to the aboveground ellipsoidal canopy volume. Using the same relationship for the volume of an individual's aboveground shoot cylinder (equation 1) and including the *shoot-root ratio* (equation 4) leads to:

$$depth_{root} = r_1 \cdot \left(\frac{sr}{f_s} \cdot B_{root}\right)^{r_2} \tag{5}$$

where the species-specific parameters r_1 and r_2 define the dependence of the rooting depth on plant biomass. Each individual has its own rooting system, irrespective of whether the individual plant has been recruited via generative or vegetative reproduction. The total branching root length $length_{root}$ [m], which is important for nitrogen uptake, is related to root biomass via the species-specific parameter *specific root length SRL* [m/g_{ODM}]:

$$length_{root} = B_{root} \cdot SRL \quad (6)$$

3 Model processes

In the following, we describe the details of the modeled processes important within the life cycle of an individual plant. These have already been introduced in brief (see chapter 1, S1.1 Fig).

3.1 Recruitment

3.1.1 Reproduction

We distinguish three different sources of species-specific recruitment of plants:

- seed rain from a surrounding meta-community
- local reproduction of plants
- sowing of seeds

Seed rain from a surrounding meta-community is modeled by a constant species-specific seed input rate N_{seed}^{meta} (in [1/m²/d], starting pre-defined at t_{meta}) while sowing throws N_{seed}^{sow} seeds (in [1/m²]) to the patch only at time t_{sow}.

In contrast, local recruitment by mother plants is dependent on their fitness. In its current version, GRASSMIND does not explicitly distinguish between vegetative and generative reproduction. Here, we assume that the single seed biomass produced via generative reproduction equals the biomass investment also required for vegetative reproduction (e.g. rhizomes or stolones). The number of locally recruited seedlings N_{seed}^{local} (in [1/m²/d]) produced by a reproductive mother plant is dependent on the mother plant's net production *NPP* allocated to its reproduction pool B_{rep} (see section 3.13) and the species-specific seed biomass B_{seed} [g_{ODM}]:

$$N_{seed}^{local} = \frac{B_{rep}}{B_{seed}} \quad (7)$$

The total number of potentially germinating seeds N_{seed} [1/m²/d] is then determined by:

$$N_{seed} = N_{seed}^{meta} + N_{seed}^{sow} + N_{seed}^{local} \quad (8)$$

3.1.2 Emergence of new seedlings

Only a limited number $\widehat{N}_{seed}$ of potential seedlings can germinate successfully within the same patch:

$$\widehat{N}_{seed} = N_{seed} \cdot germ_{\%} \quad (9)$$

where $germ_{\%}$ denotes the germination rate (unitless). In the current version of GRASSMIND, environmental conditions are not explicitly considered for the germination process, so the germination rate can be interpreted as a constant species-specific success rate.

The successfully germinated seedlings emerge dependent on an emergence time t_{em} [d] since seed rain and have an initial height h_{min} [m] (further geometrical properties can be derived from h_{min}).

At the time of emergence, seeds could additionally fail to establish in terms of limited space. If the emergence of all potentially germinating seeds would increase total vegetation cover *CC* above a patch's area (i.e. > 100%), only those seeds (for each species) proportional to the remaining free space on the patch will establish successfully (see also section 3.3.3). All seeds that failed to emerge are directly transferred to the litter pool.

3.3 Mortality

3.3.1 Senescence of leaves and root branches

All plants are subject to tissue turnover as a result of partial yellowing of leaves and the death of root branches. The transfer rate from green to yellow (senescent) shoot biomass B_{shoot}^{sen} is defined by the reciprocal value of the leaf life span *LLS* [d].

Senescent shoot leaves remain attached to an individual's shoot geometry and thus can still shade other leaves, but do not photosynthesize any longer. The transfer of senescent shoot biomass into the surface litter pool occurs either (a) if an entire plant dies or (b) at the turn of the year.

Equivalent to leaf senescence, the transfer rate of root branches into dead root biomass is defined by the reciprocal value of the root life span *RLS* [d]. Dead root parts do not remain within an individual's root system and geometry and are immediately transferred to the soil litter pool.

3.3.2 Base mortality

A base mortality is modeled using a daily constant rate m_b [1/d]. The value of this rate is a pre-defined parameter and allows differentiating dependent on the individual plant age (e.g. seedlings versus mature plants):

$$m_b = \begin{cases} 0 & ,age = 0 \\ m_{seed} & ,0 < age < age_{rep} \\ m_{basic} & ,age_{rep} \le age < life \\ 1 & ,age \ge life \end{cases} \tag{10}$$

A basic mortality rate m_{basic} is used for mature plants, whereas a special mortality rate m_{seed} is used for seedlings. Plants enter the mature plant state as soon as they start to reproduce (determined by the parameter age_{rep}). Based on the species-specific lifespan *life* [yr] and current age of plants, the rate m_b can be set to one (meaning that plants immediately die). For annual species thus m_b equals one, if the *age* [yr] of an annual plant exceeds one year. For bi-annuals and perennials m_b is set to two years or larger, respectively. Dying plants are directly transferred to the litter pool (i.e. to surface and soil litter pools for decomposition).

3.3.3 Crowding mortality

Due to space limitations only a finite number of plants are able to survive on a patch. There are different possibilities to define an indicator of limited space at which crowding mortality can be triggered.

Here, we chose an indicator M_C which is defined by the reciprocal of vegetation cover *CC* on a patch:

$$M_C = \frac{1}{CC} \tag{11}$$

If vegetation cover *CC* exceeds a patch's area, the indicator M_C drops below one. Note that vegetation cover also includes species-specific overlapping factors f_O.

Crowding mortality occurs earliest in the subsequent time step. If space is limited ($M_C < 1$), a specific number of plants N_{crowd} [1/d] die stochastically (without any species- or size-specific advantages) so that the factor M_C exceeds the threshold of one again:

$$N_{crowd} = N \cdot (1 - M_C) \tag{12}$$

Dying plants are directly transferred to the litter pool (i.e. to surface and soil litter pools for decomposition).

3.4 Light intensity

An increasing number of plants on a patch (which can differ in their plant height) results in shading among the individuals. Therefore, the global radiation I_0 [$\mu mol_{photons}/m^2/s$] on top of the highest individual is increasingly attenuated down to the bottom of the patch. To calculate vertical light conditions on a patch, the aboveground space is divided into horizontal layers of constant width Δh [m]. For each individual, its height h [m] determines the highest layer l_{max} which is covered by its shoot:

$$l_{max} = \left\lfloor \frac{h}{\Delta h} \right\rfloor \tag{13}$$

Since the leaf area L is assumed to be uniformly distributed in vertical direction within an individual's encasing cylinder, the plant's contribution of leaf area index $\hat{L}_i$ [m^2/m^2] is also assumed to be uniformly distributed among the height layers i=1,…,l_{max}:

$$\hat{L}_i = \begin{cases} \frac{L \cdot cov}{h} \cdot \Delta h & , 0 \leq i \leq l_{max} \\ 0 & , i > l_{max} \end{cases} \tag{14}$$

where cov is the ground area of the individual's encasing shoot cylinder. Summing up these leaf area contributions for all individuals on a patch results in the patch-based community leaf area index LAI_i [m^2/m^2] for each height layer i:

$$LAI_i = \frac{1}{a} \cdot \sum_{all\ individuals} k \cdot \hat{L}_i \tag{15}$$

where k denotes the species-specific light extinction coefficient and a is the area size of the patch. The light extinction coefficient is a species-specific constant parameter and includes the assumption of similar leaf angles of an individual's shoot.

To determine the irradiance I_S [$\mu mol_{photon}/m^2/s$] at the top of an individual, the patch-based leaf area indices LAI_i of all height layers above the plant's height are summed up. Light attenuation through these height layers is then calculated using the approach of Monsi and Saeki [9]:

$$I_S = I_0 \cdot e^{-\left(\sum_{i > l_{max}} LAI_i\right)} \tag{16}$$

where I_0 [$\mu mol_{photon}/m^2/s$] is the incoming photosynthetic photon flux density (*PPFD*) above canopy modeled as a daily average from sunrise to sunset.

By the calculation of the light climate within grasslands, competition for light between individuals is considered. Species that grow higher receive more light but also affect the light which is received by smaller plants via shading (S1.3 Fig). Note that not only green but also standing senescent shoot leaves contribute to shading. To reduce the effect of shading

(compared to competition between plants for belowground resources) we weight the patch-based leaf area indices LAI_i each by a factor (here 1/9, which equals a subdivision of a 1 m² patch into 9 sub-patches of homogeneous leaf area distribution). Self-shading within a single plant is included at a later stage (equation 19 and 20).

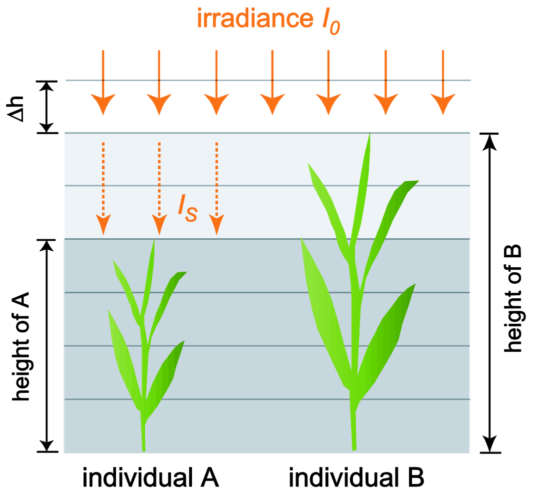

Fig S1.3. Light competition between individual species of different plant heights. Aboveground space is divided into height layers of width Δh. Each plant's leaf area is uniformly distributed among the respective covered height layers. The height layers marked in grey are shaded by plant A and B. Here, plant B is higher than plant A (vertical black arrows) and receives the unreduced incoming irradiance I_0. Plant A is shaded by those parts of plant B's leaf area that are higher than A, and hence receive the reduced irradiance I_S (equation 16).

3.5 Gross primary production

Gross biomass production of a plant is modeled via photosynthesis. Following the approach of Thornley and Johnson [10], we calculate the gross photosynthetic rate for a single leaf using a saturation function:

$$P_{leaf} = \frac{\alpha \cdot I_{leaf} \cdot p_{max}}{\alpha \cdot I_{leaf} + p_{max}} \quad (17)$$

Here, α is the species-specific initial slope of the light response curve [$\mu mol_{CO2}/\mu mol_{photon}$], p_{max} is the species-specific maximum gross photosynthetic rate [$\mu mol_{CO2}/m^2/s$], and I_{leaf} is the incoming irradiance on the leaf surface [$\mu mol_{photon}/m^2/s$]. The latter is derived by correcting the incoming irradiance I_S at the top of an individual:

$$I_{leaf} = \frac{k}{1-m} \cdot I_S \quad (18)$$

where k is the species-specific light extinction coefficient and m the transmission coefficient. To obtain the gross photosynthetic rate P_{shoot} [$\mu mol_{CO2}/m^2/s$] of an entire plant, the single-leaf photosynthesis (equation 17) is integrated over the individual's green leaf area index L_{green}:

$$P_{shoot} = \int_0^{L_{green}} P_{leaf}(\tilde{L}) d\tilde{L} \tag{19}$$

leading to:

$$P_{shoot}(I_{leaf}) = \frac{p_{max}}{k} \cdot \ln\left(\frac{\alpha \cdot k \cdot I_{leaf} + p_{max} \cdot (1-m)}{\alpha \cdot k \cdot I_{leaf} \cdot e^{-k \cdot L_{green}} + p_{max} \cdot (1-m)}\right) \tag{20}$$

Multiplying the gross photosynthetic rate (equation 20) by three conversion factors leads to the potential gross primary production GPP_{pot} [g_{ODM}/d] of a plant:

$$GPP_{pot} = P_{shoot}(I_{leaf}) \cdot \phi_{ODM} \cdot \phi_{day} \cdot \phi_a \tag{21}$$

where $\phi_{ODM} = 0.63 \cdot 44 \cdot 10^{-6}$ [$g_{ODM}/\mu mol_{CO2}$], $\phi_{day} = 60 \cdot 60 \cdot length_{day}$ [s/d] with $length_{day}$ as the number of hours per day from sunrise to sunset, and $\phi_a = cov$ [m^2].

3.7 Temperature effects

Photosynthesis and respiration are sensitive to temperature changes [11]. Gross primary production (equation 21) is reduced for air temperatures T [°C] below a threshold of 10 °C according to Schippers and Kropff ([12], see also [11], S1.4A Fig):

$$R_T = \begin{cases} 0 & , T \leq -5°C \\ 0.02857 \cdot T + 0.142 & , -5°C < T \leq 2°C \\ 0.1 \cdot T & , 2°C < T \leq 10°C \\ 1 & , 10°C < T \end{cases} \tag{22}$$

Maintenance respiration r_m increases with air temperature according to Schippers and Kropff ([12], see also [11], S1.4B Fig):

$$f_T = \begin{cases} 0 & , T \leq 0°C \\ 0.033 \cdot T & , 0°C < T \leq 15°C \\ 2^{\frac{T-25}{10}} & , 15°C < T \end{cases} \tag{23}$$

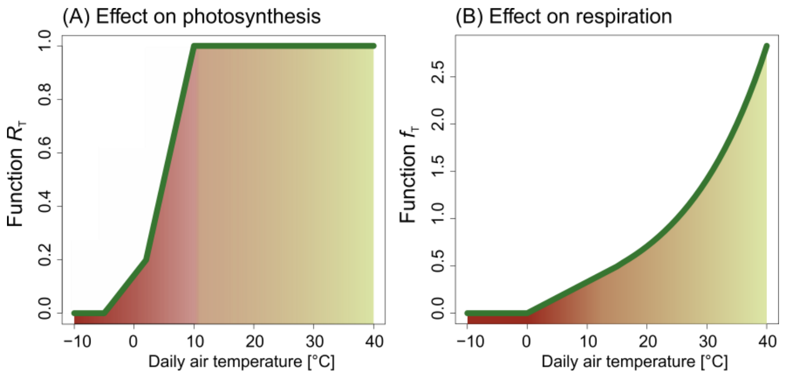

Fig S1.4. Temperature effects on (A) photosynthesis and (B) respiration of a single plant in GRASSMIND.

3.8 Water competition

The individual's uptake of water resources from soil is modeled taking into account its demand on the one hand and the soil water available on the other hand. The individual's water demand θ_{demand} [l/d], which is equal to its potential transpiration, is modeled using the water use efficiency concept:

$$\theta_{demand} = \frac{R_T \cdot GPP_{pot}}{WUE} \tag{24}$$

where GPP_{pot} [g_{ODM}/d] is the gross primary productivity, R_T (unitless) is the effect of air temperature on *GPP* and *WUE* [$g_{\text{ODM}}/kg_{\text{H2O}}$] denotes the water use efficiency (assuming 1 kg_{H2O} = 1 l_{H2O}) .

We calculate how much soil water resources are available for an individual plant although competing with other plants on a patch. By coupling GRASSMIND with soil models, the soil is divided into layers of constant width Δs for which information on soil water resources are provided. Using this vertical soil discretization, we calculate for each plant its *rooting zone* described by the soil layer s_{max} [m] (dependent on its current rooting depth):

$$s_{max} = \left\lceil \frac{depth_{root}}{\Delta s} \right\rceil \tag{25}$$

The *rooting zone* represents the composition of the respective soil layers j=1,…,s_{max} in which the individual plant is rooting (S1.5 Fig). To determine the amount of available soil water for the individual plant, we calculate the soil water content θ_W^{plant} [V%], permanent wilting point θ_{PWP}^{plant} [V%] and field capacity θ_{FC}^{plant} [V%] of the plant's *rooting zone* by summing up these variables for all layers in the rooting zone.

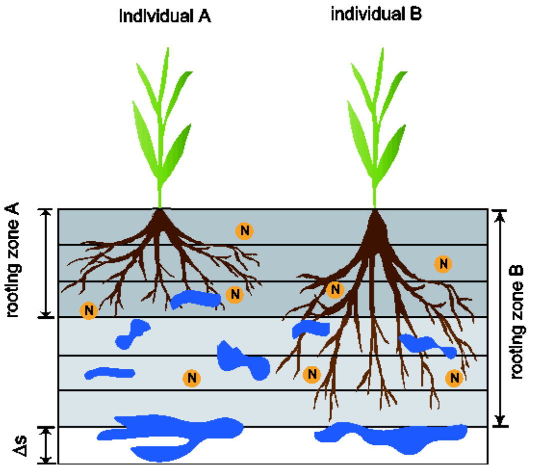

Fig S1.5. Illustration of the rooting zones of two different individuals.
Horizontal dark grey shadow marking the first three soil layers indicates the rooting zone of individual A. In this example, the rooting zone of individual B covers nearly the entire soil.

Water demand which can actually be fulfilled is determined by:

$$R_W = \begin{cases} 0 & , \theta_W^{plant} < \theta_{PWP}^{plant} \\ \frac{\theta_W^{plant} - \theta_{PWP}^{plant}}{\theta_{MSW} - \theta_{PWP}} & , \theta_{PWP}^{plant} \leq \theta_W^{plant} \leq \theta_{MSW}^{plant} \\ 1 & , \theta_{MSW}^{plant} \leq \theta_W^{plant} \end{cases} \quad (26)$$

where the factor R_W (unitless) increases from 0 to 1 (S1.6 Fig, [13]).

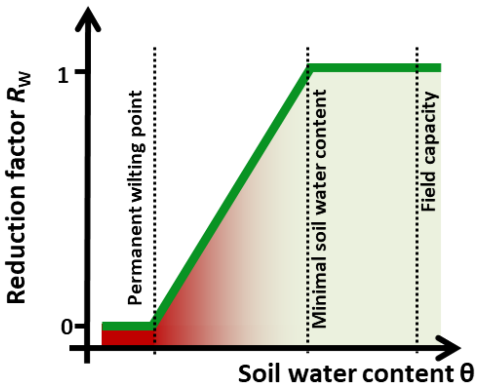

Fig S1.6. Illustration of the reduction factor R_W as a function of available soil water content.

If soil water content is below the permanent wilting point, the factor R_W is set to zero. If available soil water resources are above the *minimal soil water content* θ_{MSW}^{plant} [V%], the factor R_W is set to one:

$$\theta_{MSW}^{plant} = \theta_{PWP}^{plant} + 0.4 \cdot \left(\theta_{FC}^{plant} - \theta_{PWP}^{plant}\right) \quad (27)$$

If the soil water content is between the permanent wilting point and the minimal soil water content, soil water uptake is linearly reduced by the factor R_W. The actual water uptake θ_{uptake} [l/d] of an individual plant is calculated as follows:

$$\theta_{uptake} = R_W \cdot \theta_{demand} \quad (28)$$

For simplicity, roots are assumed to be (vertical) distributed equally among the soil layers (of the plant's *rooting zone*). Soil water uptake of an individual plant (θ_{uptake}) from a single soil layer is then calculated as:

$$\theta_{uptake}^{j} = \frac{\theta_{uptake}}{s_{max}} \quad (29)$$

where θ_{uptake}^{j} [l/d/layer] denotes the individual's water uptake from soil layer j.

Similarly, gross primary productivity of a plant GPP_{pot} [g_{ODM}/d] is reduced according to the limitation factor R_W:

$$GPP_W = R_T \cdot GPP_{pot} \cdot R_W \quad (30)$$

while GPP_W [g_{ODM}/d] is the reduced gross productivity of a plant due to water stress (R_W) and air temperature effects (R_T, see also section 3.7).

Total soil water uptake (or transpiration) of all plants on a patch can further be restricted by potential evapotranspiration (*PET* in [mm/d]) and permanent wilting point (entire soil). If the sum of transpiration of all plants (sum of θ_{uptake} for all plants on a patch in [mm/d], with 1 l_{H2O}/m^2 = 1 mm_{H2O}) and interception of rainfall by plants on a patch (*RI* in [mm/d]) exceeds the *PET*, then transpiration (or total soil water uptake $\theta_{uptake}^{patch} = \sum_{\substack{all\ plants \\ on\ patch}} \theta_{uptake}$ in [mm/d]) and gross productivity (GPP_W) of all plants is reduced proportionally by the factor (unitless):

$$f_{PET} = \frac{PET - RI}{\sum_{all\ plants} \theta_{uptake}} \quad (31)$$

Interception *RI* [mm/d] of rainfall by plants (before rainfall is percolating into the soil) is thereby calculated in the CENTURY soil model as follows:

$$RI = \left(0.0003 \cdot B_{litter}^{surface} + 0.0006 \cdot \sum_{\substack{all\ plants \\ on\ patch}} B_{shoot}\right) \cdot 0.8 \tag{32}$$

where $B_{litter}^{surface}$ [g_{ODM}/patch] is the patch's surface litter pool and B_{shoot} [g_{ODM}] is the plants' aboveground biomass.

Further, if total soil water uptake (of all plants) would result in a soil water content (θ_W^{soil} of the entire soil) below the permanent wilting point (θ_{PWP}^{soil} of the entire soil), then total soil water uptake (or transpiration θ_{uptake}^{patch}) and gross productivity (GPP_W) of all plants is reduced by the factor (unitless):

$$f_{PWP} = \frac{\theta_W^{soil} - \theta_{PWP}^{soil}}{\sum_{all\ plants} \theta_{uptake}} \tag{33}$$

3.9 Nitrogen competition

3.9.1 Nitrogen non-fixing species

Nitrogen uptake of a plant is determined dependent on the nitrogen content of the soil (N_{soil} in [g/m²]), the individual's ability to access nitrogen resources (N_{access} in [g/m²/d]) and its nitrogen demand (N_{demand} in [g/m²/d]).

To calculate N_{demand} [g/m²/d] (here, mainly from the mineral nitrogen pool of nitrate NO_3N), we calculate the amount of nitrogen required for growth of new leaves, new root branches and for seed production. For this, we calculate a preliminary net productivity NPP_{pot} (see section 3.11, based on GPP_W already constrained by soil water conditions and air temperature) and divide its carbon content by pre-defined *CN* ratios (of green and brown plant material).

$$N_{demand} = alloc_{shoot} \cdot \frac{f_C \cdot NPP_{pot}}{CN_{green}} + \left(alloc_{root} + alloc_{rep}\right) \cdot \frac{f_C \cdot NPP_{pot}}{CN_{brown}} \tag{34}$$

where $alloc_x$ are allocation rates to different carbon pools of a plant (*x* = shoot, root and reproduction). CN_{green} and CN_{root} are constant species-specific ratios of carbon to nitrogen contents in the respective pools and f_C denotes the fraction of carbon in organic dry matter (here, we use $f_C = 0.43$).

Before plants use the available soil nitrogen, we assume that their demands can be partly fulfilled by nitrogen relocated within the plant from leaves which turn from green to senescent (section 3.14). If demands cannot be fulfilled by relocated nitrogen from senescent leaves, the

remaining demand is covered by soil nitrogen resources (for which plants have to compete with each other).

The amount of soil nitrogen N_{access} which is potentially available for use by an individual plant is calculated by:

$$N_{access} = \sum_{j<s_{max}} F_j \cdot N_j \quad (35)$$

where N_{access} [g/m²/d] denotes the available nitrogen in soil to which the plant has access and F_j represents a root competition factor. The factor F_j regulates the amount of nitrogen an individual can access by competing with other individuals. F_j is defined as the percentage of root branch lengths of the plant in relation to total root branch length (of all plants per soil layer *j*):

$$F_j = \frac{\left(\frac{length_{root}}{s_{max}}\right)}{\left(\sum_{\substack{all\,plants\\ on\ patch\\ with\ j<s_{max}}} \left(\frac{length_{root}}{s_{max}}\right)\right)} \quad (36)$$

We assume that the plant's total root branches $length_{root}$ are distributed equally among the soil layers (in its *rooting zone*, S1.5 Fig). The potential nitrogen uptake N_{uptake} [g/m²/d] is then defined as:

$$N_{uptake} = \min(N_{demand}, N_{access}) \quad (37)$$

Net production of a plant can be reduced due to limitation in available soil nitrogen (see section 3.11). Therefore, we introduce a limitation factor R_N which is calculated by:

$$R_N = \frac{N_{uptake}}{N_{demand}} = \min\left(1, \frac{N_{access}}{N_{demand}}\right) \quad (38)$$

3.9.2 Nitrogen fixing species

Symbiotic nitrogen fixation, e.g. by legumes, is modeled using the following assumptions:

- N-fixing species never compete for nitrogen with other plants. Thus, the limitation factor R_N always equals one.

- Plants loose carbon for uptake of nitrogen via symbiosis. A specific fraction $rhiz_{\%}$ of net primary production *NPP* is therefore provided to rhizobia which is not available anymore for structural growth or recruitment.

In GRASSMIND positive effects of nitrogen-fixing species occur as those species do not take part in the nitrogen competition process and thus, more nitrogen resources in soil are available for N-non-fixing plants. Further, all species contribute to soil nitrogen via decomposition after plant death.

3.10 Respiration

We consider respiratory costs for maintenance of structural tissue (shoot and root biomass) and for growth of plants. Maintenance costs r_{main} [g_{ODM}/d] are assumed to be proportional to the green shoot biomass B_{shoot}^{green} and living root biomass B_{root}:

$$r_{main} = r_m \cdot f_T \cdot \left(B_{shoot}^{green} + B_{root}\right) \quad (39)$$

where r_m is a constant maintenance respiration rate [1/d] and the factor f_T accounts for changing demands for maintenance respiration with varying air temperature (see section 3.7). Growth respiratory costs are modeled by a constant parameter r_g (unitless).

3.11 Net primary production

A plant's gross primary production is used for (a) respiratory costs, (b) growth of an individual's shoot and root and (c) reproduction. Losses due to respiration (maintenance and growth) lead to the individual's net primary production NPP_{act} [g_{ODM}/d]:

$$NPP_{act} = R_N \cdot \left(1 - r_g\right) \cdot (GPP_{act} - r_{main}) \quad (39)$$

where GPP_{act} [g_{ODM}/d] is the actual gross primary production which can be reduced according to environmental limitations or competition (derived from the potential GPP_{pot}):

$$GPP_{act} = f_{PET} \cdot f_{PWP} \cdot R_W \cdot R_T \cdot GPP_{pot} \quad (40)$$

where R_W, f_{PET}, f_{PWP}, R_N, and R_T (all ranging in [0,1]) account for reductions due to soil water limitations (R_W, f_{PET}, f_{PWP}, equations 26, 31 and 33), soil nitrogen limitation (R_N, equation 38), and temperature effects (R_T, equation 22), respectively.

3.13 Allocation of net primary production

Net primary production NPP_{act} (equation 39) is allocated to (a) shoot biomass, (b) root biomass and (c) to reproduction as follows:

- the fraction allocated to the shoot ($alloc_{\mathrm{shoot}}$) is a species-specific parameter
- the fraction allocated to the root ($alloc_{\mathrm{root}}$) is derived from the fixed species-specific shoot-root ratio sr (equation 4)

$$alloc_{root} = \frac{alloc_{shoot}}{sr} \quad (41)$$

- the remaining fraction is allocated to the reproduction pool

$$alloc_{rep} = 1 - alloc_{shoot} - alloc_{root} \quad (42)$$

For seedlings, the fraction allocated to the reproduction pool is zero (when plant age is below age_{rep}). Hence, the fraction allocated to the shoot is adjusted as follows:

$$alloc_{shoot} = \frac{sr}{sr+1} \quad (43)$$

3.14 Growth of a plant

Allocation of produced biomass (dependent on the allocation fractions, section 3.13) and senescence (dependent on leaf life span LLS and root life span RLS, section 3.3.1) result in the following changes in the plant's biomass pools:

$$\frac{\Delta B_{shoot}^{sen}}{\Delta t} = \frac{1}{LLS} \cdot B_{shoot}^{green} \quad (44)$$

$$\frac{\Delta B_{shoot}^{green}}{\Delta t} = alloc_{shoot} \cdot NPP_{act} - \frac{1}{LLS} \cdot B_{shoot}^{green} \quad (45)$$

$$\frac{\Delta B_{root}}{\Delta t} = alloc_{root} \cdot NPP_{act} - \frac{1}{RLS} \cdot B_{root} \quad (46)$$

$$\frac{\Delta B_{rep}}{\Delta t} = alloc_{rep} \cdot NPP_{act} \quad (47)$$

Based on the change in the biomass pools B_{shoot} and B_{root}, the new biomass values for the different pools are calculated and the geometrical variables of an individual plant are updated.

With regard to a plant's biomass pools, the respective nitrogen pools are updated dependent on the species-specific CN ratios (of green and brown plant material) and a biomass-to-carbon conversion factor (f_{C} = 0.43). For those shoot biomass parts, which turn from green to

senescent ones (equation 44), leaf nitrogen is relocated and used for nitrogen demands in the subsequent time step (section 3.9.1):

$$N_{relocated} = \frac{1}{LLS} \cdot B_{shoot}^{green} \cdot f_C \cdot \left(\frac{1}{CN_{green}} - \frac{1}{CN_{brown}}\right) \quad (48)$$

3.15 Management

Management of grasslands is included in GRASSMIND in terms of mowing, irrigation and fertilization. All three events are characterized by the following information:

- dates of management events (i.e. frequency)
- intensity (e.g. cutting height, fertilizer amount, amount of water supply)

In terms of mowing, the height of all plants on a patch greater than the cutting height (e.g. 10 cm) is decreased, thus leading to a modified parameter $hw_{modified}$. For the following time steps, increment in aboveground biomass only contributes to height growth until the time step at which the original parameter *hw* is reached again.

Fertilizer and water supply by irrigation contribute to soil resources in the upper soil layer.

References

1. Taubert F, Frank K, Huth A. A review of grassland models in the biofuel context. Ecological Modelling. 2012;245:84-93.

2. Fischer R, Bohn F, Dantas de Paula M, Dislich C, Groeneveld J, Gutiérrez AG, et al. Lessons learned from applying a forest gap model to understand ecosystem and carbon dynamics of complex tropical forests. Ecological Modelling. 2016;326:124-33.

3. Botkin DB, Janak JF, Wallis JR. Some ecological consequences of a computer model of forest growth. J Ecol. 1972;60(3):849-73.

4. Köhler P, Huth A. Simulating growth dynamics in a South-East Asian rainforest threatened by recruitment shortage and tree harvesting. Climatic Change. 2004;67(1):95-117.

5. Shugart HH. Terrestrial ecosystems in changing environments. Cambridge: Cambridge University Press; 1998 1998.

6. Franko U, Oelschlägel B, Schenk S. Simulation of temperature, water and nitrogen dynamics using the model CANDY. Ecological Modelling. 1995;81(1-3):213-22.

7. Parton WJ, Stewart JWB, Cole CV. Dynamics of C, N, P and S in grassland soils: a model. Biogeochemistry. 1988;5(1):109-31.

8. Schenk HJ, Jackson RB. Rooting depths, lateral root spreads and below-ground/above-ground allometries of plants in water-limited ecosystems. Journal of Ecology. 2002;90(3):480-94.

9. Monsi M, Saeki T. The light factor in plant communities and its significance for dry matter production. Japanese Journal of Botany. 1953;14(1):22-52.

10. Thornley JHM, Johnson IR. Plant and Crop Modelling. Oxford: Claredon Press; 1990 1990.

11. Larcher W. Ökologie der Pflanzen. Verbesserte Auflage. Verlag Eugen Ulmer; 1976.

12. Schippers P, Kropff MJ. Competition for light and nitrogen among grassland species: a simulation analysis. Functional Ecology. 2001;15(2):155-64.

13. Granier A, Breda N, Biron P, Villette S. A lumped water balance model to evaluate duration and intensity of drought constraints in forest stands. Ecol modell. 1999;116(2-3): 269-283.
