# Supplementary material for: Confronting an individual-based simulation model with empirical community patterns of grasslands
Source: PLoS One. 2020 Jul 28;15(7):e0236546. doi: 10.1371/journal.pone.0236546 (PMC7386574; doi:10.1371/journal.pone.0236546)
Supplement: S2 Appendix — (PDF) [file pone.0236546.s002.pdf]

S2 Appendix

Additional information on empirical data and input parameter of the grassland model GRASSMIND

Empirical data and derivation of model parameter

We used published empirical data on a biodiversity grassland experiment carried out in Central Germany (Jena Experiment, Germany, 50°55´N, 11°35´E, [1]). We selected three species (*Festuca pratensis*, *Poa pratensis*, *Plantago lanceolata*) for the parameterization of the grassland model GRASSMIND. For each species, a monoculture plot (20 m x 20 m) is available, and one two-species-mixture plot (*P. pratensis* and *P. lanceolata*).

Observed vegetation attributes

The available measurements include empirical data for seven consecutive years (starting in year 2002). From 2003 onwards, plots have been censused biannual in terms of (a) aboveground biomass (AGB), (b) leaf area index (LAI), (c) vegetation height, and (d) vegetation cover [2]. We focused only on the six-year data (2003 to 2008) and calculated the arithmetic mean and standard deviation of both censuses per year to derive annual values.

Replicated measurements per census within the plot are available for aboveground biomass (3 to 4 replicates) and vegetation height (10 replicates). In both cases, we used the median value of all replicates for each of the bi-annual censuses.

Because weed cover was considerably high in some censuses (and the grassland model does not simulate weeding), we handled the cover of observed weeds as additional potential space of the target species (as if weeds were not present) and therefore, corrected the measured species cover by adding the respective observed weed cover.

Sowing and seed rain

Seeds of each monoculture and the mixture were sown on bare field in the period 11-16 May 2002. For the grassland model, we assumed a sowing date of 16 May 2002. In general, seeds might also disperse from the surrounding landscape to the experimental plots (and even across the plots). To account for this, we summarized seeds which had been sown and dispersed from the surrounding landscape in one model parameter. We allowed different model parameter values of seed rain for the four field plots.

Mowing

The field plots were mown twice a year to a height of 10 cm (which is included in the simulation model). In the first year, plots were mown twice, eight weeks after sowing (in July) and in September [3] (we used: 11 July and 20 September). In all consecutive years (2003 to 2008), plots were mown twice a year in June and September [2] (we used: 20 June and 20 September).

Climate

Climatic conditions for the local study site (precipitation, global radiation, relative humidity and air temperature) were daily measured by two weather stations located near the experiment. Data from January 2004 onwards are supplied by the weather station of the Max Planck Institute (MPI, [4]) for Biogeochemistry in Jena, Germany. Missing climate data for the years 2002 and 2003 were substituted by data from the weather station of the Ernst-Abbe-Fachhochschule Jena (FH Jena, [5]). Gaps of missing data at three days were filled with the mean value of the previous and following day. Potential evapotranspiration was derived from air temperature, global radiation and relative humidity [6], while day length was calculated based on the latitude [7].

Soil properties

In the Century soil model, we modelled three soil horizons: (i) the upper 10 cm soil layer, (ii) 10 cm – 30 cm soil layer and (iii) 30 cm – 200 cm soil layer. Soil properties differ between these soil layers (S2.1 Table). For each plot, empirical data on (a) soil texture (silt, sand and clay content), (b) permanent wilting point (PWP), (c) field capacity (FC), (d) mineral nitrogen content and (e) dry bulk density was available. Measurements are available

in different soil layers (different for each property; down to 30 cm soil). We calculated the average soil property for those soil layers which match the defined layers in the Century model (S2.1 Table). In case of no available data (e.g. for the third soil layer below 30 cm soil depth) we used the same properties as for the second soil layer. No data on the initial soil water content of each plot was available. We therefore used a gradient from 0% to 100% of relative soil water (ranging between PWP and FC) across the defined soil layers (S2.1 Table). Bulk density was only used for deriving mineral nitrogen content per soil. Because measured bulk density was only available for the monoculture plot of *F. pratensis*, we used the same values per soil layer also for the other plots.

Table S2.1. Summary of soil properties per experimental plot and soil layer.

**Plot**<br>(code and sown species)	**Soil layer (cm)**	**Relative soil water content**	**Field capacity (V%)**	**Permanent wilting point (V%)**	**Bulk density (g/cm³)**	**Mineral nitrogen content (g/m²)**	**Texture (%) (silt/sand/clay)**
**Mixture**<br>B1A16<br>(*P. pratensis* + *P. lanceolata*)	0 - 10<br>10 - 30<br>30 - 200	0<br>0.5<br>1	27.6<br>28<br>28	14.3<br>14.9<br>14.9	NA	0.12<br>0.45<br>3.73	50/30/20
**Monoculture**<br>B2A05<br>(*F. pratensis*)	0 - 10<br>10 - 30<br>30 - 200	0<br>0.5<br>1	30.1<br>31.3<br>31.3	17<br>17.7<br>17.7	1.06<br>1.41<br>1.37	0.23<br>0.97<br>8.01	56/22/22
**Monoculture**<br>B2A13<br>(*P. lanceolata*)	0 - 10<br>10 - 30<br>30 - 200	0<br>0.5<br>1	30.9<br>31.9<br>31.9	17.9<br>19.3<br>19.3	NA	0.13<br>0.47<br>3.87	56/22/22
**Monoculture**<br>B4A12<br>(*P. pratensis*)	0 - 10<br>10 - 30<br>30 - 200	0<br>0.5<br>1	33.2<br>33.3<br>33.3	20.7<br>20.8<br>20.8	NA	0.20<br>0.47<br>3.94	69/8/23

Codes for each experimental plot are given as denoted in the published data sets of the grassland experiment [2].

Model parameterization

Cost function for inverse model parameterization

The cost function for the optimization included the comparison of all derived vegetation patterns of time-series data between observation and simulation. For a selected pattern *p* (AGB, LAI, vegetation height and cover) and species *i*, we calculated the mean

absolute percentage error (*MAPE*, [8]) of the simulated ($y_{p,i}$) and observed ($x_{p,i}$) time-series data (of sample size T_n):

$$\boldsymbol{C_{mono}} = \sum_{\substack{pattern \\ p=1}}^{4} \sum_{\substack{species \\ i=1}}^{3} \sum_{\substack{time\ point \\ j=1}}^{T_n} \frac{\mathbf{1}}{\boldsymbol{T_n}} \left(\frac{|y_{p,i,j} - x_{p,i.j}|}{x_{p,i,j}} \right)$$

We used the cost function C_{mono} for optimizing the monoculture plots of the three species. For two species (*P. pratensis* and *P. lanceolata*) we had extended observations of a two-species mixture, which we also included in the optimization by a second cost function C_{mix}.

$$\boldsymbol{C_{mix}} = \sum_{\substack{pattern \\ p=1}}^{2} \sum_{\substack{time\ point \\ j=1}}^{T_n} \frac{\mathbf{1}}{\boldsymbol{T_n}} \left(\frac{|y_{p,i,j} - x_{p,i.j}|}{x_{p,i,j}} \right) + \sum_{\substack{pattern \\ p=3}}^{4} \sum_{\substack{species \\ i=1}}^{2} \sum_{\substack{time\ point \\ j=1}}^{T_n} \frac{\mathbf{1}}{\boldsymbol{T_n}} \left(\frac{|y_{p,i,j} - x_{p,i.j}|}{x_{p,i,j}} \right)$$

For the patterns of vegetation height and LAI (p = 1, 2), only measurements of the community were available, while for AGB and cover (p = 3, 4) detailed measurements per species could be used.

In addition, we calculated for the grass-forb mixture the time-series of relative yields *RY* in terms of aboveground biomass and coverage. Therefore, we divided the (simulated or observed) value in the mixture by the (simulated or observed) value in the monoculture ([9]) for both species separately:

$$RY(y_{p,i}) = \frac{y_{p,i,mix}}{y_{p,i,mono}}, \quad RY(x_{p,i}) = \frac{x_{p,i,mix}}{x_{p,i,mono}}$$

whereby $y_{p,i,mix}$describes the simulated data point of pattern p and species i in the mixture plot, $y_{p,i,mono}$ denotes the simulated data point of pattern p and species i in the monoculture plot and $x_{p,i,mix}$ and $x_{p,i,mono}$ represent the corresponding observed data points.

Again, we calculated for each species and each of both time-series of relative yields (of AGB and vegetation cover) the *MAPE* value between simulation and observation and calculated the cost function C_{RY} similar to the other ones:

$$C_{RY} = \sum_{\substack{pattern \\ p=3}}^{4} \sum_{\substack{species \\ i=1}}^{2} \sum_{\substack{time\ point \\ j=1}}^{T_n} \frac{1}{T_n} \left(\frac{|y_{p,i,j} - x_{p,i.j}|}{x_{p,i,j}} \right)$$

Including relative yields in the calibration additionally weights on species interactions within the calibration process.

We applied the '*dynamically dimensioned search*' optimization algorithm with 5,000 steps [10], which searched for an optimal set of model parameters by minimizing the total cost function $C = C_{mono} + C_{mix} + C_{RY}$. In each optimization step, the grassland model GRASSMIND was simulated and averaged for nine replicates (to account for stochasticity) ~~on 9 m²~~ with the algorithmic chosen parameter set, starting from bare ground in 2002 (seeding year) and simulating until the end of 2008 (the first year 2002 is neglected in the cost function). Initial height of seedlings is predefined at 3 cm from which other state variables (e.g. shoot biomass) are derived dependent on the species traits (see S1 Appendix for details). See S2~~10~~ Fig for the illustration of the different optimization steps and successful minimizations.

We decided for the applied algorithm due to a lower computational runtime (of about 4 days for 5,000 steps for GRASSMIND) compared to longer runtimes of other approaches (e.g. Approximate Bayesian Computation (ABC) techniques, [11,12]). Although ABC techniques allow to assess also parameter uncertainties, our modeling study focuses mainly on asking whether in general an optimal model parameter set can be found in order to reproduce the observed grassland dynamics.

Model evaluation criteria

Model evaluation is based on the comparison of all derived vegetation patterns of time-series data between observation and simulation (~~of GRASSMIND~~ average of 100 replicated GRASSMIND model simulations to account for stochasticity; ~~on 100 m²~~ based on the previously determined parameterization). For a selected pattern *p* and species *i*, we

performed a linear regression of the simulated ($y_{p,i}$) and observed ($x_{p,i}$) time-series data (which resulted in a slope s, intercept I and coefficient of determination R^2):

$$y_{p,i} = I_{p,i} + s_{p,i} \cdot x_{p,i}$$

In contrast to the intercept $I_{p,i}$, the slope $s_{p,i}$ and the coefficient of determination R^2 are dimensionless measures irrespective of the analyzed pattern. As patterns differ in their value ranges (e.g. aboveground biomass in the order of 100 g/m², but leaf area index in the order of 1 m²/m²), we normalized the intercept $I_{p,i}$ by the median of observed and simulated time-series data for a given pattern p and species i. We used the median in contrast to the mean or maximum in order to ensure reasonable values also if data are not Gaussian distributed (or having a high variation).

In addition, the normalized root mean square error ($nrmse_{p,i}$) per pattern was included (using the standard deviation σ of the observed time-series data for normalization):

$$nrmse_{p,i} = \sqrt{\sum_{\substack{\text{census} \text{time point} \\ j=1}}^{T_N N} \frac{\left(y_{p,i,j} - x_{p,i,j}\right)^2}{T_N N}} / \sigma(x_{p,i})$$

To calculate the *nrmse*, we used the R package hydroGOF [13]. See Table S2.2 and S3~~11~~ Fig for explanations.

Table S2.2. Summary of model evaluation criteria.

Evaluation criteria		Name	Unit	Range of values	Optimal value	Meaning
Linear regression between observed and simulated time-series (per pattern p and species i)	I	Normalized intercept (by the median of observed and simulated data)	-	[0; ∞)	0	Measure of reproducing observed data quantitatively by the model (hint to systematic errors of the simulation model)
	s	Regression slope	-	(-∞;∞)	1	Measure of reproducing observed data trends qualitatively by the model
	R^2	Coefficient of determination	-	[0; 1]	1	Measure of variation of simulated data
	$nrmse$	Normalized root mean square error	%	[0; ∞)	0	Measure of explicit differences between observation and simulation

The table reports for each criterion its unit, range of values, optimal value and a brief description of its meaning.

Results of the inverse model parameterization and evaluation

For the monocultures we received an optimized cost function of C_{mono} = 2.72 (N = (6 years × 3 species × 4 patterns)), while for the two-species mixture cost were lowered to C_{mix} = 1.65 (N = (6 years × 2 patterns) + (6 years × 2 species × 2 patterns)). The additional cost function in terms of relative yields for the species mixtures was similarly around C_{RY} = 1.18 (N = (6 years × 2 species × 2 patterns)). In total, we got a cost function after optimization of C = 5.55. Details on the inverse parameterization are listed in S2.3 Table and resulting model parameters are shown in Table 1 and Table 2.

Table S2.3. Comparison of observed and simulated patterns in terms of aboveground biomass, vegetation height and cover and leaf area index for monoculture plots, a two-species mixture plot and corresponding relative yields.

**Plot**	**Species**	**Pattern**	**Observed**	**Simulated**	***MAPE***	***Regression* (Intercept *I*, slope *s*, R^2)**	***nrmse***
**Mono-culture**	*F. pratensis*	AGB (g m^{-2})	219.6 (111.8 – 327.5)	158.7 (139.8 – 177.5)	0.38	I = 0.89<br>s = 0.07<br>R^2 = 0.07	97.5%
		Height (cm)	36.1 (27.1 – 45.2)	32.2 (27.0 – 37.4)	0.15	I = 0.59<br>s = 0.35<br>R^2 = 0.40	78.2%
		LAI (-)	2.0 (1.6 – 2.4)	1.9 (1.6 – 2.1)	0.16	I = 0.46<br>s = 0.49<br>R^2 = 0.31	86.2%
		Cover (%)	61.8 (56.1 – 67.5)	74.1 (70.7 – 77.6)	0.53	I = 1.01<br>s = 0.005<br>R^2 = 0.001	106.6%
	P. pratensis	AGB (g m^{-2})	88.8 (44.3 – 133.4)	70.2 (55.9 – 84.4)	0.48	I = 1.0<br>s = 0.01<br>R^2 = 0.01	100.1%
		Height (cm)	20.2 (18.8 – 21.5)	17.8 (14.2 – 21.5)	0.12	I = 0.82<br>s = 0.16<br>R^2 = 0.22	92.3%
		LAI (-)	1.0 (0.8 – 1.2)	0.8 (0.6 – 1.0)	0.16	I = 0.77<br>s = 0.18<br>R^2 = 0.73	97.7%
		Cover (%)	74.5 (72.7 – 76.3)	69.7 (61.1 – 78.4)	0.28	I = 0.91<br>s = 0.04<br>R^2 = 0.05	92.2%
	P. lanceolata	AGB (g m^{-2})	67.4 (49.3 – 85.5)	55.4 (40.0 – 70.7)	0.32	I = 0.92<br>s = 0.07	93.4%

					R^2 = 0.33	
		Height (cm)	18.7<br>(16.8 – 20.6)	15.0<br>(11.0 – 19.0)	0.22	I = 0.78<br>s = 0.19<br>R^2 = 0.45	90.9%
		LAI (-)	1.2<br>(0.9 – 1.6)	1.1<br>(0.8 – 1.4)	033	I = 0.97<br>s = 0.03<br>R^2 = 0.03	93.3%
		Cover (%)	55.0<br>(45.1 – 64.9)	70.1<br>(64.2 – 76.0)	0.39	I = 1.13<br>s = -0.11<br>R^2 = 0.25	152.7%
**Mixture**	*P. pratensis*	AGB (g m^{-2})	53.2<br>(36.5 – 70.0)	27.8<br>(20.8 – 34.8)	0.46	I = 0.84<br>s = 0.04<br>R^2 = 0.09	104.1%
	P. lanceolata	AGB (g m^{-2})	88.5<br>(50.2 – 126.8)	37.9<br>(29.7 – 46.2)	0.45	I = 0.57<br>s = 0.19<br>R^2 = 0.87	106.8%
	Community	Height (cm)	20.3<br>(16.8 – 23.8)	18.2<br>(14.6 – 21.9)	0.12	I = 0.97<br>s = 0.01<br>R^2 = 0.003	101.9%
	Community	LAI (-)	1.3<br>(0.9 – 1.7)	1.0<br>(0.8 – 1.3)	0.20	I = 1.02<br>s = -0.02<br>R^2 = 0.0006	134%
	P. pratensis	Cover (%)	32.3<br>(28.1 – 36.6)	28.8<br>(26.7 – 31.0)	0.27	I = 0.7<br>s = 0.22<br>R^2 = 0.22	85.2%
	P. lanceolata	Cover (%)	44.9<br>(36.5 – 53.3)	41.1<br>(35.9 – 46.3)	0.32	I = 0.95<br>s = 0.05<br>R^2 = 0.06	92.7%
**Relative yields**	*P. pratensis*	AGB	0.55<br>(0.37 – 0.73)	0.39<br>(0.37 – 0.41)	0.44	I = 0.74<br>s =0.14<br>R^2 = 0.35	397.6%
	P. lanceolata	AGB	1.31<br>(0.89 – 1.73)	0.69<br>(0.65 – 0.73)	0.43	I = 0.68<br>s = -0.09<br>R^2 = 0.05	340.8%
	P. pratensis	Cover	0.45<br>(0.38 – 0.51)	0.41<br>(0.39 – 0.44)	0.20	I = 0.63<br>s = 0.31<br>R^2 = 0.43	156.1%
	P. lanceolata	Cover	0.88<br>(0.74 – 1.01)	0.59<br>(0.56 – 0.61)	0.43	I = 0.99<br>s = -0.05<br>R^2 = 0.07	733.8%

Compared patterns are aboveground biomass (AGB), vegetation height and cover, and leaf area index (LAI) for three species monocultures and a two-species mixture (average of yearly mean values; in brackets the average of yearly minimum and maximum values is given). Per selected pattern the mean absolute percentage error (*MAPE*), the normalized *nrmse*, the regression slope, normalized intercept and the coefficient of determination are given.

References

1. Weisser W. et al. Biodiversity effects on ecosystem functioning in a 15-year grassland experiment: Patterns, mechanisms, and open questions. Basic and Applied Ecology 2017;23: 1-73.

2. Weigelt A, Marquard E, Temperton VM, Roscher C, Scherber C, Mwangi PN, et al. The Jena Experiment: six years of data from a grassland biodiversity experiment. Ecology. 2010;91(3):930-1.

3. Heisse K, Roscher C, Schumacher J, Schulze ED. Establishment of grassland species in monocultures: different strategies lead to success. Oecologia. 2007;152(3):435-47.

4. Max-Planck-Institute for Biogeochemistry, URL: https://www.bgc-jena.mpg.de/wetter/ [last accessed: 5 July 2019]

5. Ernst-Abbe-Hochschule Jena, University of Applied Sciences, Klimatologische Messstation, URL: http://wetter.mb.fh-jena.de/station/index.html [last accessed: 5 July 2019].

6. Turc L. Estimation of irrigation water requirements, potential evapotranspiration: a simple climatic formula evolved up to date. Ann. Agron 1961;12(1), 13-49.

7. Forsythe WC, Rykiel Jr EJ, Stahl RS, Wu HI, Schoolfield RM. A model comparison for daylength as a function of latitude and day of year. *Ecological Modelling.* 1995;80: 87-95.

8. De Myttenaere A, Golden B, Le Grand B, Rossi F. Mean absolute percentage error for regression models. *Neurocomputing*. 2016;192: 38-48.

9. Loreau M, Hector A. Partitioning selection and complementarity in biodiversity experiments. Nature. 2001;412(6842):72-6.

10. Lehmann S, Huth A. Fast calibration of a dynamic vegetation model with minimum observation data. Ecological Modelling. 2015;301:98-105.

11. Beaumont MA. Approximate Bayesian computation in evolution and ecology. *Annual review of ecology, evolution, and systematics*. 2010;41: 379-406.

12. Hartig F, Calabrese JM, Reineking B, Wiegand T, Huth A. Statistical inference for stochastic simulation models–theory and application. *Ecology letters*. 2011;14(8): 816-827.

13. Zambrano-Bigiarini M. hydroGOF: Goodness-of-fit functions for comparison of simulated and observed hydrological time series. *R package version 0.3-10*. 2017. Available from: http://hzambran.github.io/hydroGOF/.<doi:10.5281/zenodo.840087>.
