# Supplementary figures and images for: Confronting an individual-based simulation model with empirical community patterns of grasslands
Source: PLoS One. 2020 Jul 28;15(7):e0236546. doi: 10.1371/journal.pone.0236546 (PMC7386574; doi:10.1371/journal.pone.0236546)

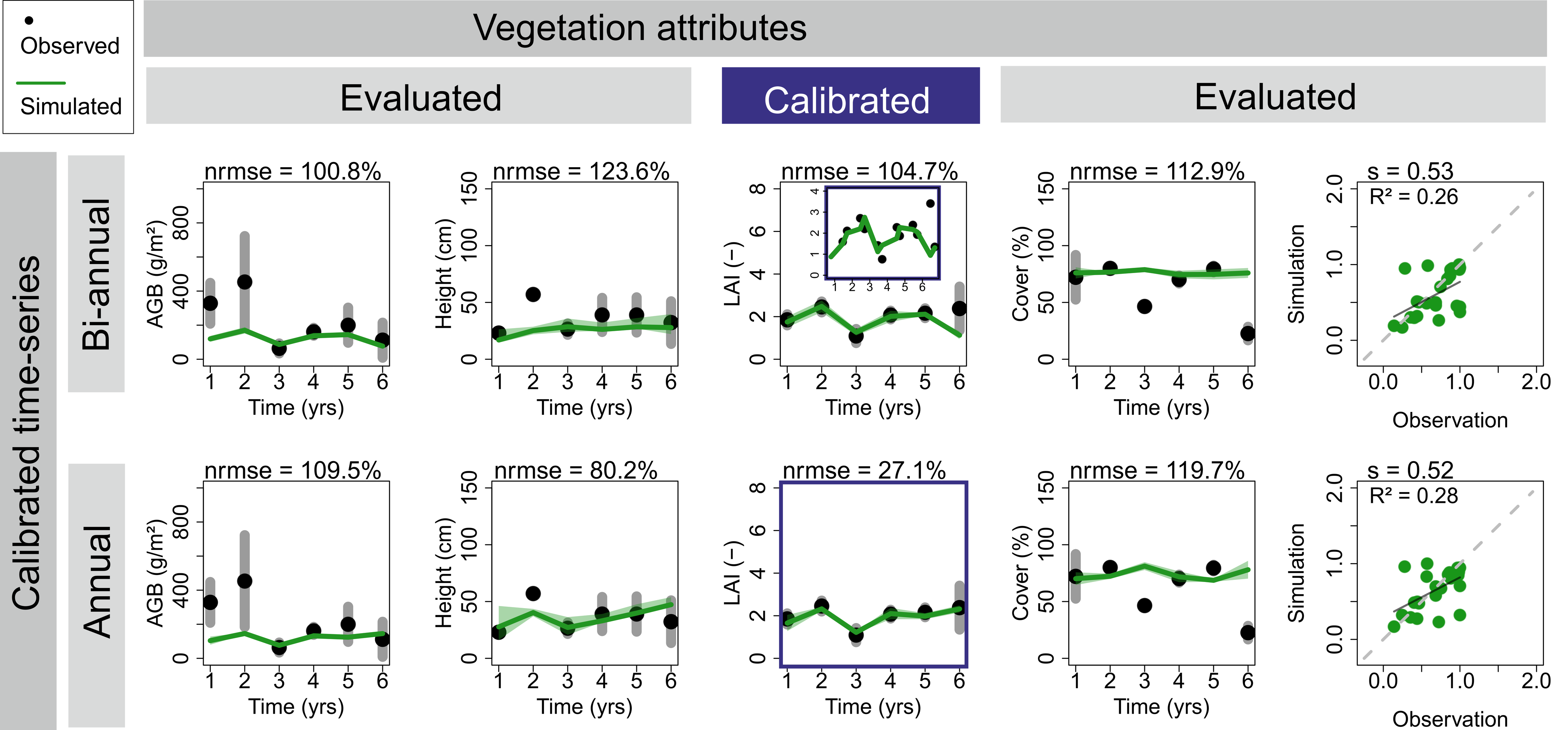

Supplement: S1 Fig — The example of F. pratensis monoculture is shown here. The calibrated vegetation pattern (yearly mean values versus bi-annual measurements) is framed by a blue rectangle (for bi-annual shown as inlet) while the other vegetation patterns are shown for evaluation purposes. All four vegetation patterns are normalized and summarized in a 1:1 plot (right panel). (TIF) [file pone.0236546.s003.tif]

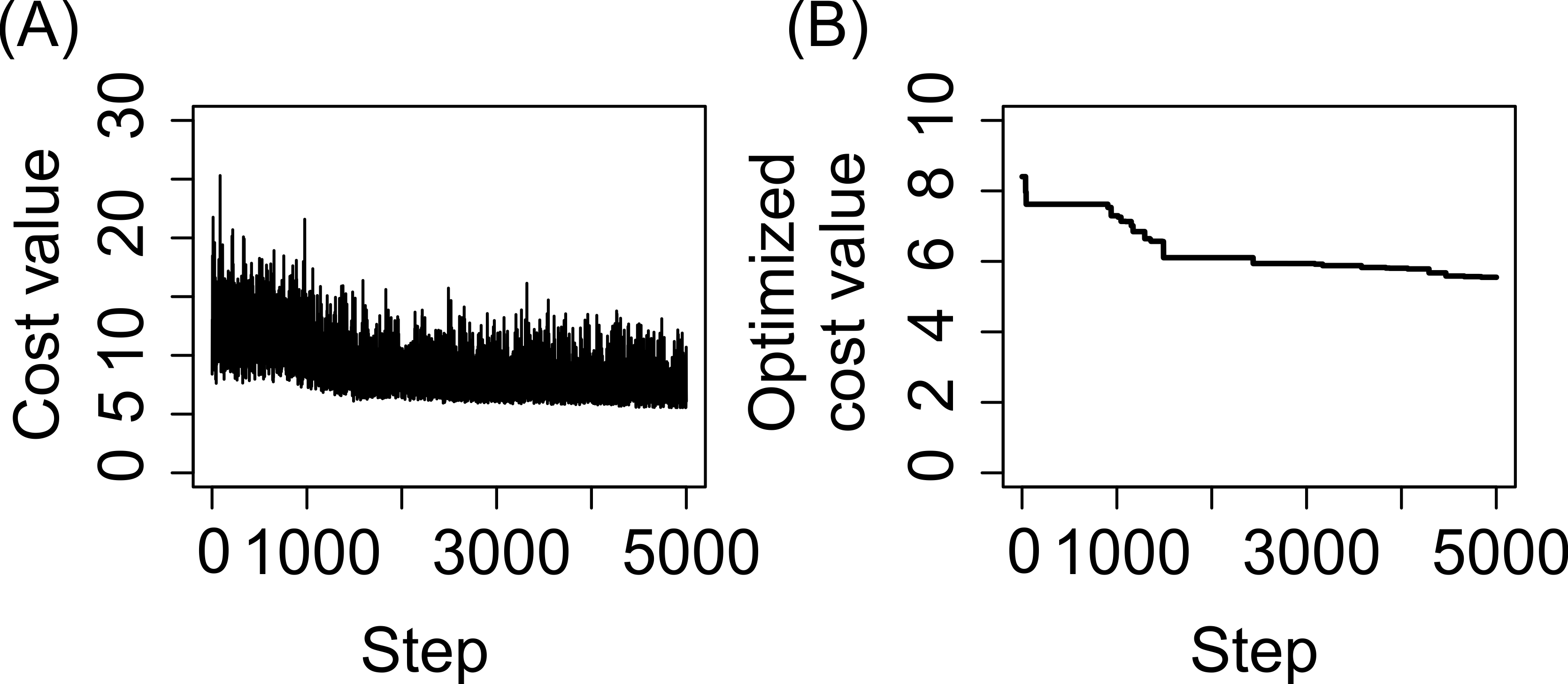

Supplement: S2 Fig — In (A) each optimization and the corresponding cost function is shown (using MAPE as cost function and the DDS algorithm for optimization). In (B) only successfully minimized optimization steps are displayed. See methods for details. (TIF) [file pone.0236546.s004.tif]

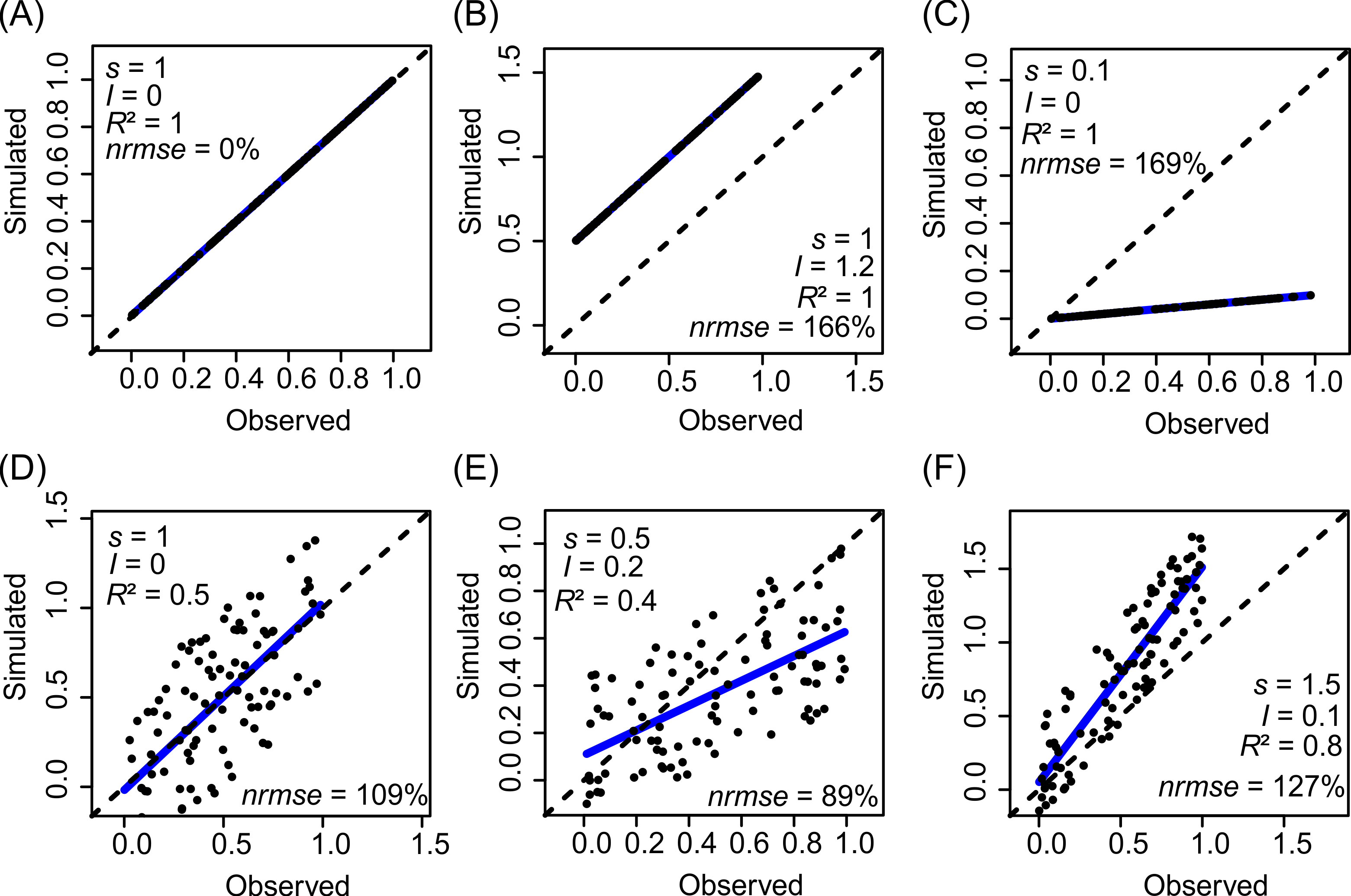

Supplement: S3 Fig — Examples of comparisons between virtually created observed and simulated data points (black dots) to illustrate the different evaluation criteria (the linear regression line is visualized in blue). In (A) virtual simulations match perfectly virtual observations. In (B) the qualitative trend of observations is reproduced perfectly by simulations (indicated by slope s = 1 and R2 of 1) while the positive intercept and nrmse shows a systematic deviation. In (C) simulations and observations deviate from each other (reflected by the low R2 and high nrmse) but reproduce on average the qualitative and quantitative trends (due to intercept of I = 0 and slope of s = 1). In (D) and (E) examples which do not fulfill any criteria optimally are shown. In each panel, the 1:1 grey dotted line illustrates optimal evaluation criteria. (TIF) [file pone.0236546.s005.tif]

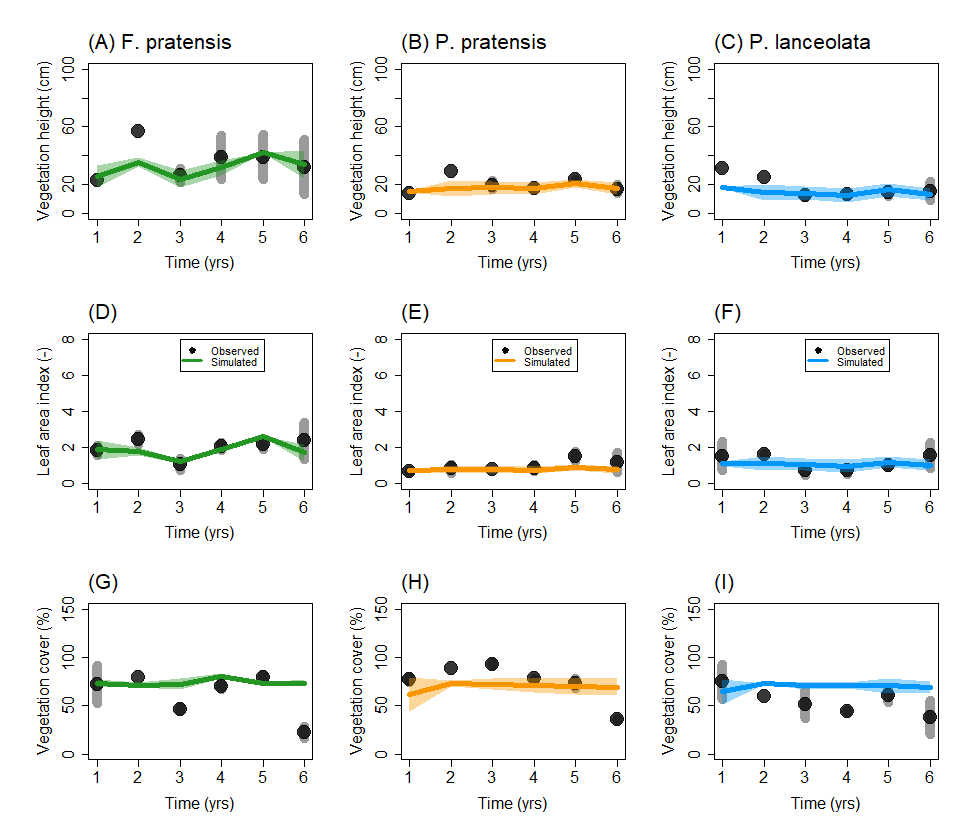

Supplement: S4 Fig — Black dots show observed values and colored lines represent the simulated dynamics using GRASSMIND (black dots/colored lines show the annual average and vertical grey lines/polygons denote the range of two single measurement values per year). (TIF) [file pone.0236546.s006.tif]

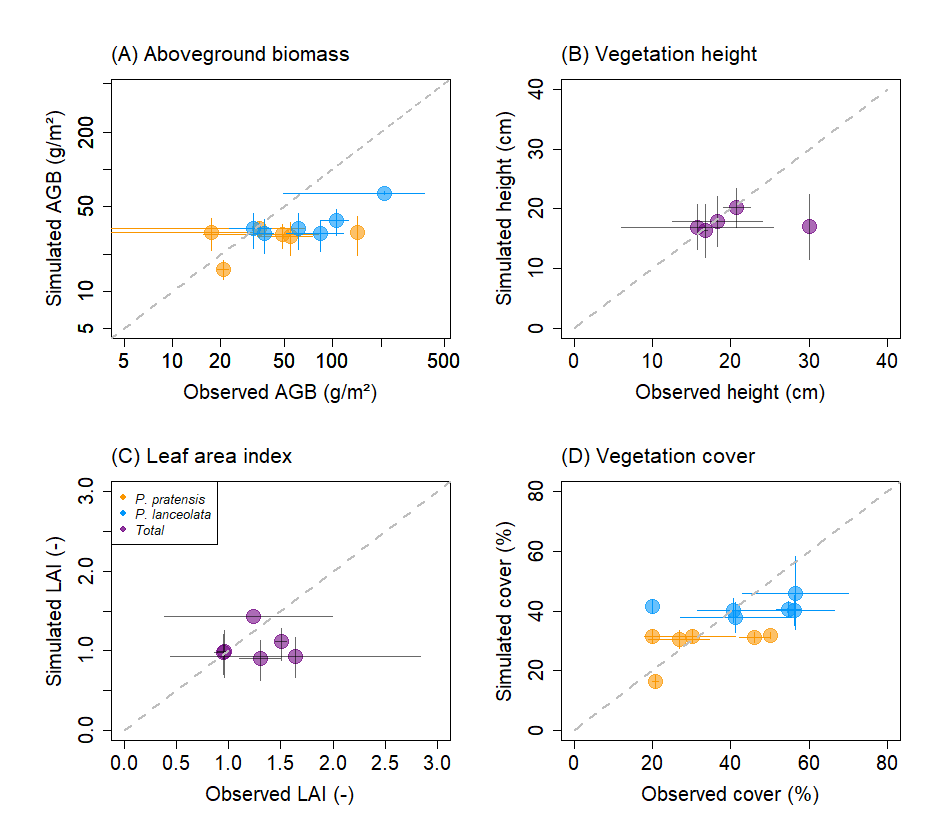

Supplement: S5 Fig — Each dot reflects the comparison of an observed with a simulated yearly value of a selected pattern. Colors identify the two species (orange–P. pratensis, blue–P. lanceolata, purple–total community). (TIF) [file pone.0236546.s007.tif]

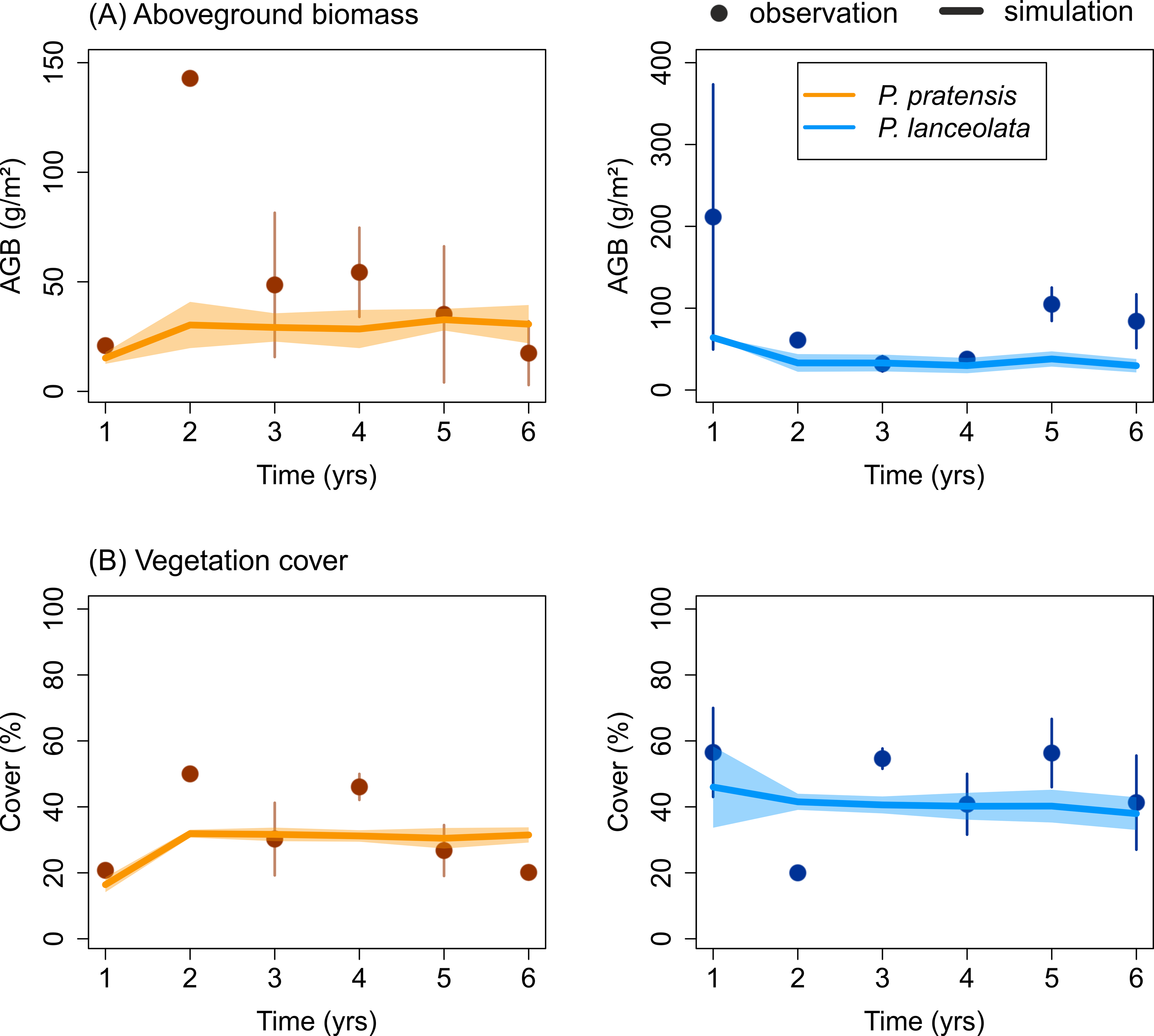

Supplement: S6 Fig — Dynamics of (A) aboveground biomass and (B) vegetation cover for the species mixture. Dots show observed values and colored lines represent the simulated dynamics using GRASSMIND (both show the annual average and vertical grey lines/polygons denote the range of two census values per year). (TIF) [file pone.0236546.s008.tif]

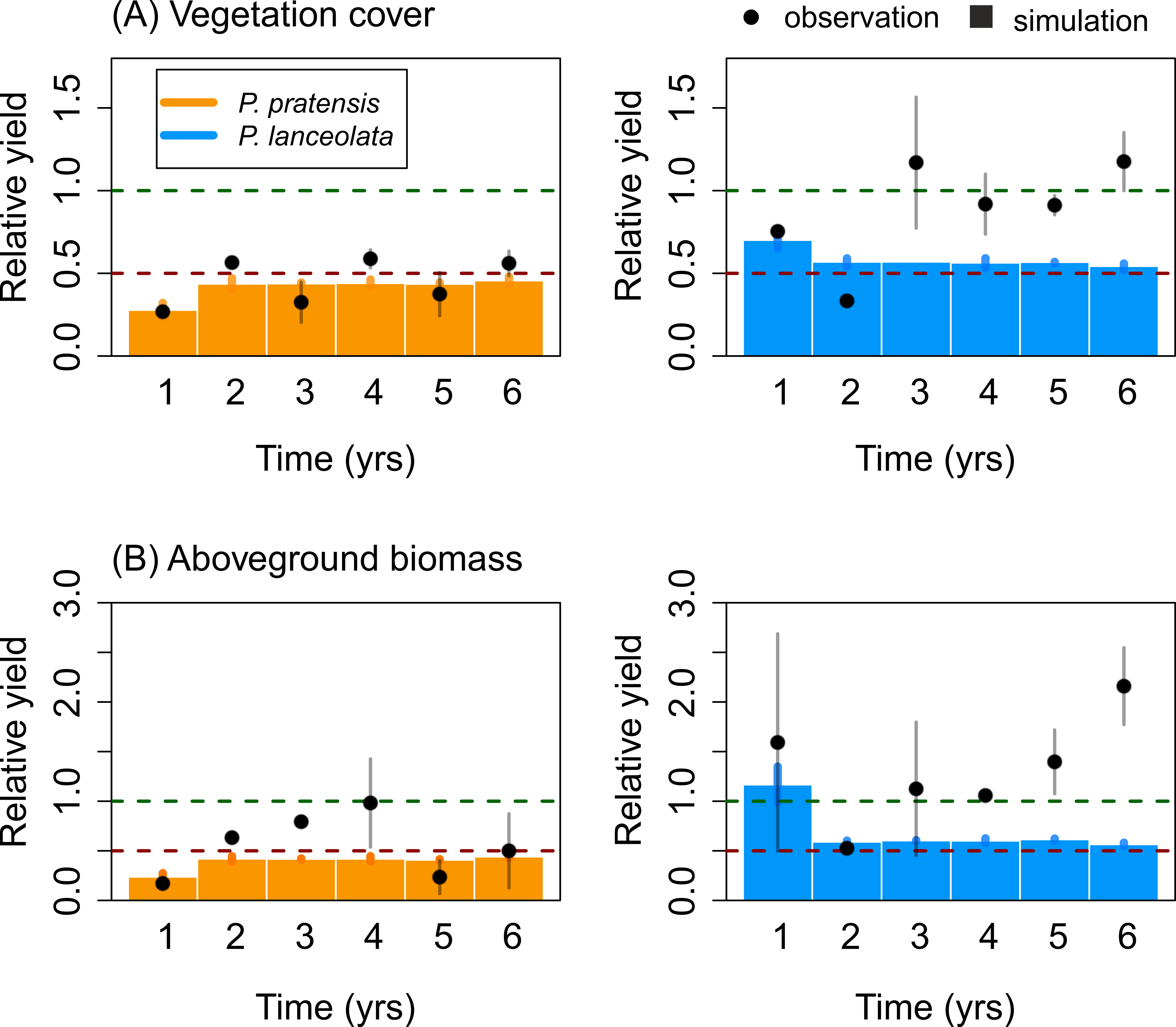

Supplement: S7 Fig — Relative yield of (A) vegetation cover and (B) aboveground biomass for two species included in the mixture. Bars show the simulated mixture performance of a species divided by its monoculture performance. Black dots show the analogous empirical data. A relative yield of 1 (green dotted line) means that the species behaves in the mixture similar compared to its monoculture (although in competition for space and resources with the other species). The red dotted line (value of 0.5) represents the expected relative yield of a species in the mixture (assuming both species equally distribute resources among them). (TIF) [file pone.0236546.s009.tif]

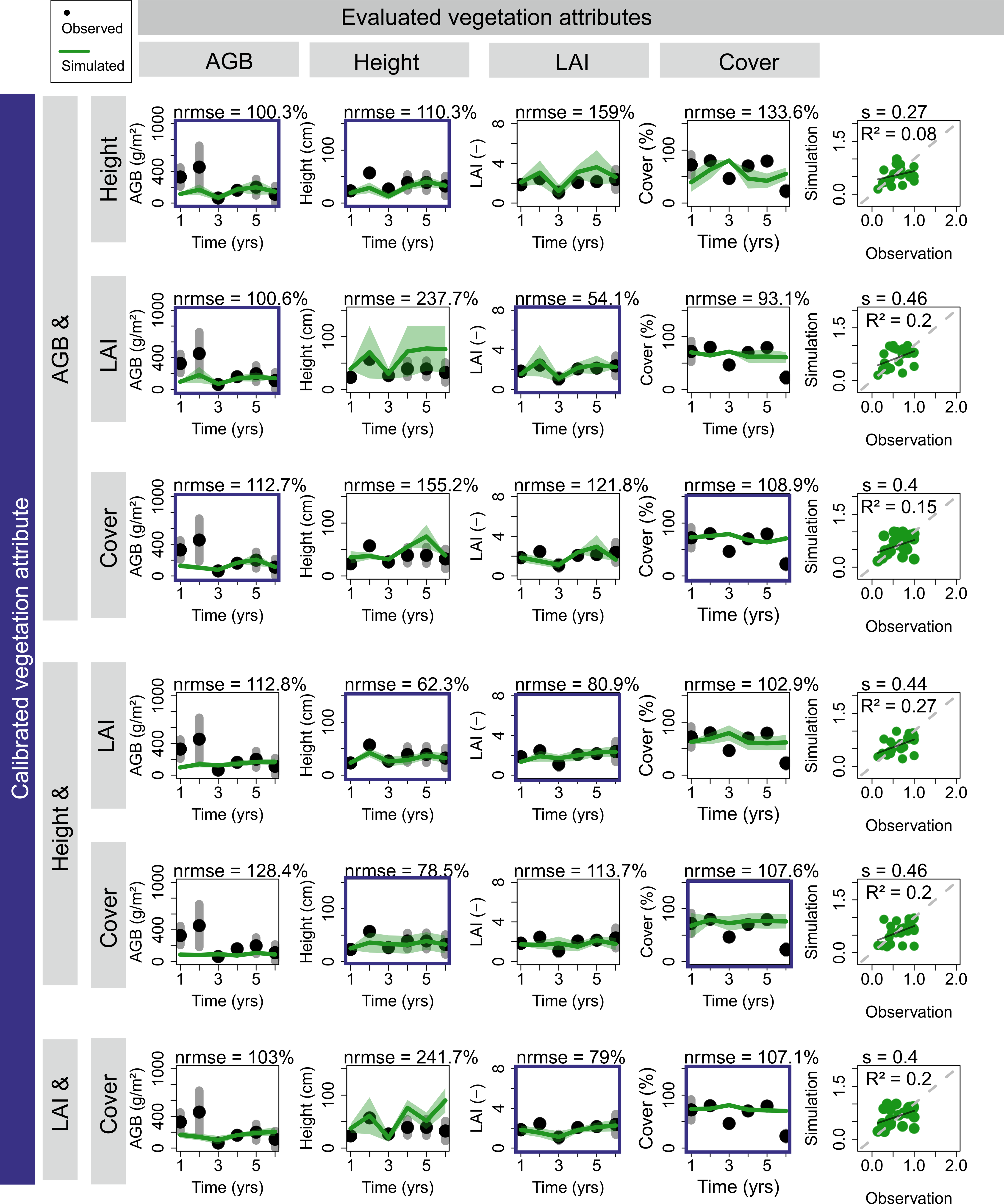

Supplement: S8 Fig — The calibrated vegetation patterns are framed by a blue rectangle while the other vegetation patterns are shown for evaluation purposes (example of F. pratensis monoculture, using MAPE as cost function). Green lines (and shaded polygons) describe simulations (yearly mean and range) while black dots and grey lines describe the observations (yearly mean and range). All four vegetation patterns are normalized and summarized in a 1:1 plot (right panel). (TIF) [file pone.0236546.s010.tif]

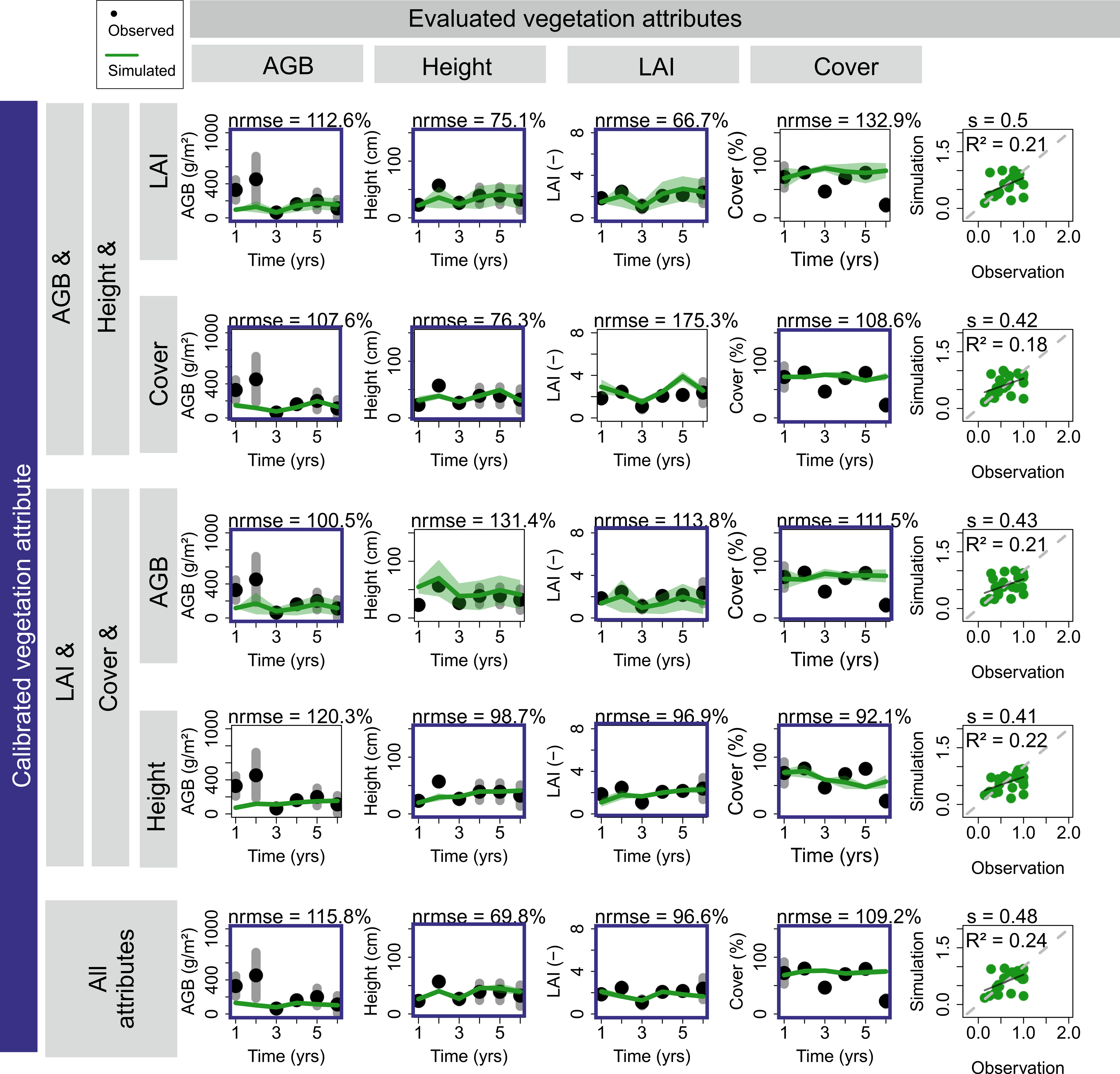

Supplement: S9 Fig — The calibrated vegetation patterns are framed by a blue rectangle while the other vegetation patterns are shown for evaluation purposes (example of F. pratensis monoculture, using MAPE as cost function). Green lines (and shaded polygons) describe simulations (yearly mean and range) while black dots and grey lines describe the observations (yearly mean and range). All four vegetation patterns are normalized and summarized in a 1:1 plot (right panel). (TIF) [file pone.0236546.s011.tif]

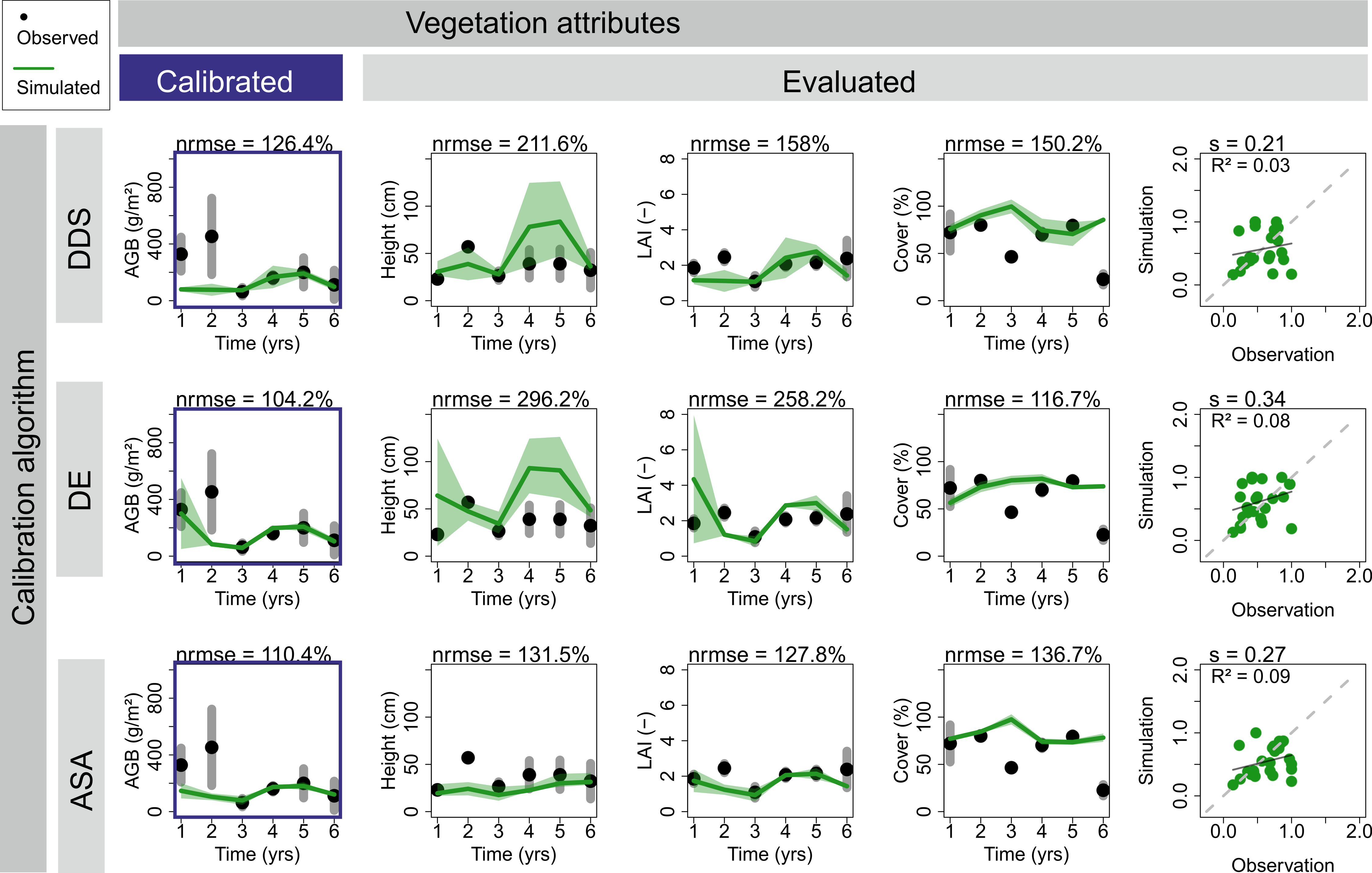

Supplement: S10 Fig — The calibrated vegetation pattern (here on the example of AGB) is framed by a blue rectangle while the other vegetation patterns are shown for evaluation purposes. Green lines (and shaded polygons) describe simulations and black dots (with grey lines) the observations (yearly mean and annual range). All four vegetation patterns are normalized and summarized in a 1:1 plot (right panel). (TIF) [file pone.0236546.s012.tif]

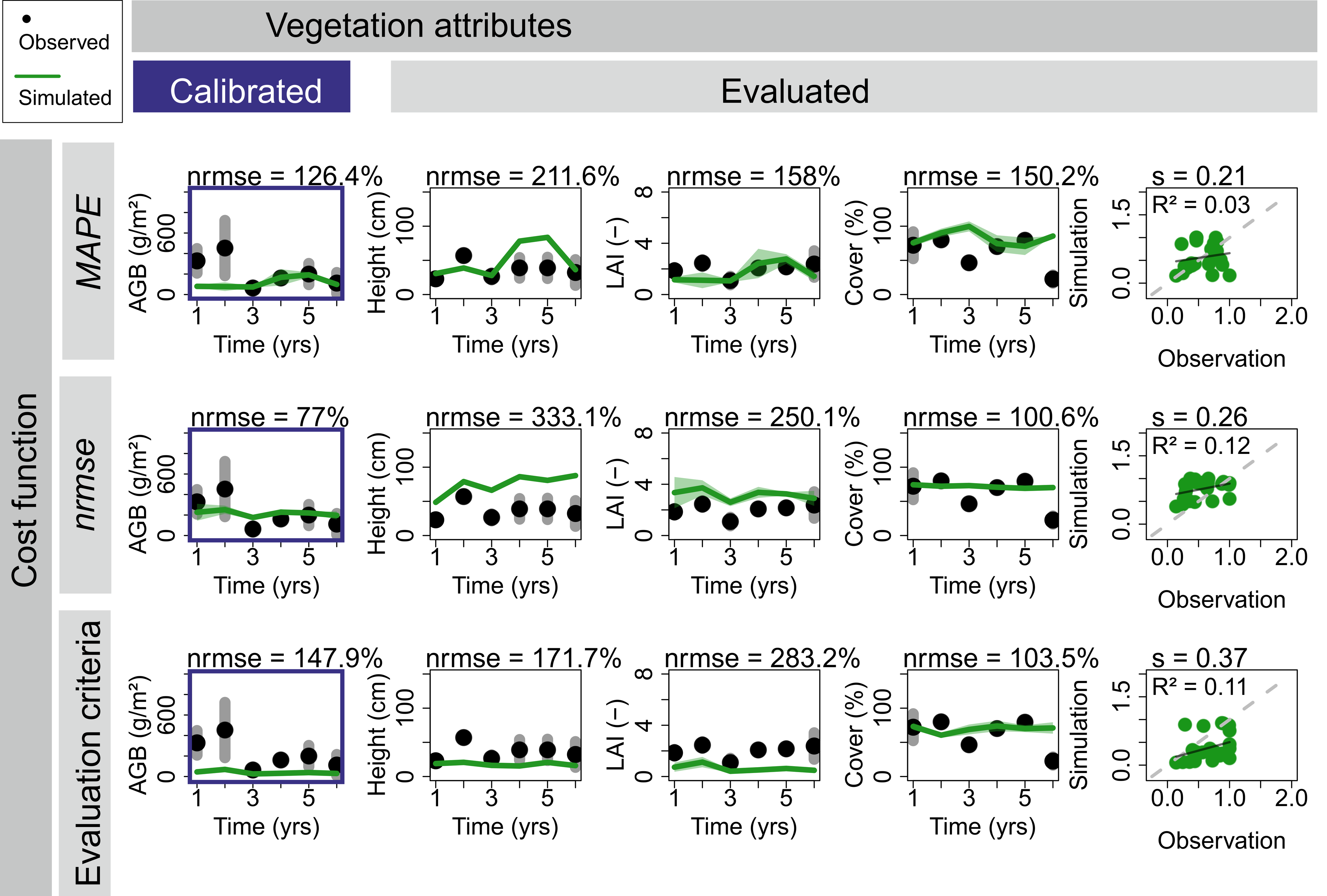

Supplement: S11 Fig — The calibrated vegetation pattern (here on the example of AGB) is framed by a blue rectangle while the other vegetation patterns are shown for evaluation purposes. Green lines (and shaded polygons) describe simulations and black dots (with grey lines) the observations (yearly average and annual range). All four vegetation patterns are normalized and summarized in a 1:1 plot (right panel). (TIF) [file pone.0236546.s013.tif]
